# A novel super-enhancer-related gene signature predicts prognosis and immune microenvironment for breast cancer

**DOI:** 10.1186/s12885-023-11241-2

**Published:** 2023-08-18

**Authors:** Qing Wu, Xuan Tao, Yang Luo, Shiyao Zheng, Nan Lin, Xianhe Xie

**Affiliations:** 1 Department of Oncology, Molecular Oncology Research Institute, The First Affiliated Hospital of Fujian Medical University, No. 20 Chazhong Road, Fuzhou, 350005 China; 2 Department of Oncology, National Regional Medical Center, Binhai Campus of The First Affiliated Hospital, Fujian Medical University, Fuzhou, 350212 China; 3 Department of Pathology, The First Affiliated Hospital of Fujian Medical University, Fuzhou, China; 4 College of Clinical Medicine for Oncology, Fujian Medical University, Fuzhou, Fujian China; 5 Fuzong Clinical Medical College of Fujian Medical University, Fuzhou, Fujian China; 6 Department of Gastrointestinal Surgery, The 900th Hospital of Joint Logistics Support Forces of Chinese PLA, Fuzhou, Fujian China; 7 Fujian Key Laboratory of Precision Medicine for Cancer, The First Affiliated Hospital, Fujian Medical University, Fuzhou, 350005 China

**Keywords:** Breast cancer, Super-enhancer, Overall survival, Tumor immune microenvironment, Immune checkpoints

## Abstract

**Background:**

This study targeted at developing a robust, prognostic signature based on super-enhancer-related genes (SERGs) to reveal survival prognosis and immune microenvironment of breast cancer.

**Methods:**

RNA-sequencing data of breast cancer were retrieved from The Cancer Genome Atlas (TCGA), 1069 patients of which were randomly assigned into training or testing set in 1:1 ratio. SERGs were downloaded from Super-Enhancer Database (SEdb). After which, a SERGs signature was established based on the training set, with its prognostic value further validated in the testing set. Subsequently, we identified the potential function enrichment and tumor immune infiltration of the model. Moreover, in vitro experiments were completed to further explore the biological functions of ZIC2 gene (one of the risk genes in the prognostic model) in breast cancer.

**Results:**

A risk score system of prognostic value was constructed with 6 SERGs (ZIC2, NFE2, FOXJ1, KLF15, POU3F2 and SPIB) to find patients in high-risk group with significantly worse prognosis in both training and testing sets. In addition, a multivariate regression was established via integrating the 6 genes with age and N stage, indicating well performance by calibration, time-dependent receiver operating characteristic (ROC) analysis and decision curve analysis (DCA). Further analysis demonstrated that tumor-associated pathological processes and pathways were significantly enriched in the high-risk group. In general, the novel SERGs signature could be applied to screen breast cancer with immunosuppressive microenvironment for the risk score was negatively correlated with ESTIMATE score, tumor-infiltration lymphocytes (such as CD4 + and CD8 + T cell), immune checkpoints and chemotactic factors. Furthermore, down-regulation of ZIC2 gene expression inhibited the cell viability, cellular migration and cell cycle of breast cancer cells.

**Conclusions:**

The novel SERGs signature could predict the prognosis of breast cancer; and SERGs might serve as potential therapeutic targets for breast cancer.

**Supplementary Information:**

The online version contains supplementary material available at 10.1186/s12885-023-11241-2.

## Introduction

Breast cancer has been the most common global malignancy as well as the leading cause of cancer deaths [1]. During the past recent years, various therapies have emerged in the era of breast cancer. However, there are still a large portion of breast cancer patients with poor prognosis and high mortality due to its heterogeneity and the complexity of cancer pathogenesis, development, and metastasis [2, 3]. Studies have shown that status of estrogen receptor (ER), progesterone receptor (PR), human epidermal growth factor receptor 2 (HER2), circulating tumor cell DNA (ctDNA), carcinoembryonic antigen (CEA), carbohydrate antigen 15−3 (CA15-3), extracellular vesicles (EV), circulating miRNA, BRCA gene mutations and other biomarkers were closely related to diagnosis and prognosis of breast cancer, but without specificity and stability [4–10]. Therefore, it is critical to find appropriate prognostic and predictive biomarkers for breast cancer.

In 2013, Richard A. Young and his colleagues firstly proposed the concept of super-enhancers (SEs), which were a class of cis-regulatory elements with super transcriptional activation characteristics [11, 12], with broad application prospects in the discovery of pathogenic driver genes, the analysis of the susceptibility of disease-associated variation sites, the development of accurate diagnosis as well as drug development of complex disease [13, 14]. In recent years, accumulating studies have focused on whether SEs could enable the identification of effective biomarkers in cancer and the development of therapeutics targeting transcriptional addiction [15–17]. Therefore, SE-related genes (SERGs) are expected to be potential biomarkers for predicting prognosis and anti-tumor therapy [18, 19]. Nevertheless, the robust relationship between SEs and breast cancer remains to be further clarified [20, 21]. Hence, it is urgent to explore the potential molecular mechanisms and prognostic indicators of breast cancer based on SEs.

In this study, we obtained RNA-sequence and clinicopathological data of breast cancer patients from The Cancer Genome Atlas (TCGA) database. Then, through a comprehensive bioinformatics analysis, we established a risk score system with 6 prognosis SERGs and validated it by testing set. Meanwhile, enrichment analyses were conducted between the low-risk and high-risk groups to reveal the potential mechanisms and pathways. In addition, analyses based on tumor infiltration immune level, immune checkpoints and chemotactic factors were applied to investigate the associations between SERGs signature and tumor immune microenvironment (TIME). Furthermore, reverse transcription quantitative polymerase chain reaction (RT-qPCR), Western Blot, immunohistochemistry (IHC) and functional experiments (such as Cell Counting Kit-8 (CCK-8) assay, cellular migration assay and cell cycle assay) were all adopted to validate the expression level and significant clinical value of ZIC2 (one of the risk genes in the prognostic model) in cell lines and tissues of breast cancer.

## Materials and methods

### Data Acquisition

Complying with the TCGA data access policies and publication guidelines, gene expression profile (RNA-sequencing) with corresponding clinical information of breast cancer and normal samples were obtained from the publicly-available TCGA database (https://portal.gdc.cancer.gov/) [22].

### Identification of SERGs

A total of 153 SERGs were identified from SEdb 2.0 (www.licpathway.net/sedb/) through the following screening process: human, NCBI GEO/SRA, tissue, mammary gland (Sample_02_0667; Sample_02_0670; Sample_02_0671; Sample_02_1517) after removal of duplicates (the transcriptional abundance of 153 SERGs in TCGA-BRCA as shown in Additional File 1).

### Selection and functional clustering analyses of SERGs

|log2 (fold change) | value of > 1 and false discovery rate (FDR) control (adjusted *P* < 0.05) were set as the cut-offs to screen for differentially expressed genes (DEGs) between tumor and normal samples. Venn analysis was utilized to select overlapping genes, and Volcano plots of the DEGs were generated using the “ggplot2” package (https://ggplot2.tidyverse.org). Furthermore, the “cluster Profiler” package and Metascape database (https://metascape.org/gp/#/main/step1) were applied to conduct Gene Ontology (GO) and Kyoto Encyclopedia of Genes and Genomes (KEGG) analyses for patients based on the SERGs [23]. Additionally, Search Tool for the Retrieval of Interacting Genes (STRING) (http://string-db.org) database was adopted to predict protein-protein interaction (PPI) network of significantly positive DEGs and analyze the degree of interactions between proteins [24].

### Construction and validation of a Novel SERGs signature

Univariate Cox analysis of overall survival (OS) was conducted to screen for SERGs with prognostic value and visualized by forest plots (*P* < 0.05) [25]. A total of 1069 breast cancer patients were randomly assigned into training or testing set in 1:1 ratio for constructing and validating the SERGs signature. Subsequently, the Least Absolute Shrinkage and Selection Operator (LASSO) regression was performed with tenfold cross-validation and a *P* value of 0.05 based on the training set [26]. Finally, 6 SERGs were integrated into a risk signature, and the risk score of every breast cancer patient was calculated according to the normalized expression level of SERGs and corresponding regression coefficients, with the following computational formula:

1 $$\displaylines{Risk{\text{ }}score{\text{ }} = \sum\limits_{i = 1}^n {\exp ressio{n_{gene\_i}}} \times \cr lasso\_coeffieicen{t_{gene\_i}} \cr}$$

After that, patients in training set were divided into low-risk and high-risk groups with the median value of risk score as the threshold value, and the OS between groups were compared by Kaplan-Meier (KM) analysis. Then, time-dependent receiver operating characteristic (ROC) curve analysis was performed with “survivalROC” R package to evaluate the predictive accuracy of the SERGs signature. In order to validate the prognostic model, the risk score for every breast cancer patient in testing set was also calculated in accordance with the same formula as training set, and patients in testing set were further divided into low-risk and high-risk groups according to the median value of risk score (median value of the testing set was different from the threshold from the training set).

DNA methylation plays a key role in prognostic assessment and potential biomarkers for cancer development [27]. In this study, MethSurv (https://biit.cs.ut.ee/methsurv/) was adopted to determine the expression and prognostic patterns of single CpG methylation of the SERGs in breast cancer [28], with the DNA methylation values represented by beta values ranging from 0 to 1.

### Risk score and clinicopathological characteristics

Breast cancer patients in training set were also assigned into subgroups by clinicopathological characteristics [including age, T stage, N stage, M stage, TNM stage, status of ER, PR, HER2, and menopause (Pre: <6 months since last menstrual period AND no prior bilateral ovariectomy AND not on estrogen replacement; Post: prior bilateral ovariectomy OR > 12 months since last menstrual period with no prior hysterectomy)]. Then, the associations between risk score and clinical features were identified, and the predictive value of SERGs signature in subgroups were also presented based on various clinical characteristics.

### Nomogram and Validation

Univariate and multivariate Cox regression analyses were conducted with clinicopathological indicators (including age, T stage, N stage, M stage, status of ER, PR, HER2 and menopause). Then, integrating the risk score with age and N stage, a multi-variate regression was established by a nomogram to predict the OS rates of 1-, 3- and 5-year. In addition, calibration, time-dependent ROC analysis, concordance index (C-index) and decision curve analysis (DCA) for model were completed to assess the discriminatory ability of the nomogram.

### DEGs between risk groups

The “edgeR” R package was used to identify the DEGs between high-risk and low-risk groups with FDR < 0.05 and |log2FC| ≥1. GO and KEGG analyses with *P* value < 0.05 were considered statistically significant. Then, GSEA (http://software.broadinstitute.org/gsea/index.jsp) (version 3.14.3) [29] was adopted to investigate the hallmarks of high-risk group and get visualized by ridge map.

### Associations between SERGs signature and TIME

The immune infiltration scores and tumor-infiltrating immune cells (TIICs) of breast cancer samples in training set were calculated by ESTIMATE [30] and Immune Cell Abundance Identifier (ImmuCellAI) (http://bioinfo.life.hust.edu.cn/web/ImmuCellAI/) [31], respectively. In addition, we also validated the correlation between identified genes (SERGs) and immune cells by means of the “TIMER” (http://timer.cistrome.org/) analysis tool [32]. Meanwhile, the associations between SERGs signature and immune checkpoints and chemotactic factors were also analyzed to further predict the TIME of breast cancer.

### Preliminary Experimental Verification for ZIC2

The mRNA expression level of ZIC2 in various breast cancer cell lines was validated by Cancer Cell Line Encyclopedia (CCLE) (https://sites.broadinstitute.org/ccle) and RT-qPCR, and the protein expression level of ZIC2 in cell lines and tissues was displayed by Western Blot and IHC. Referring to the Human Protein Atlas (HPA) database (https://www.proteinatlas.org/), immunocytochemistry (ICC) images were also obtained to detect and visualize ZIC2 protein in the human HEK293 cell line and U251MG glioblastoma cell line by antibodies specific to the target. In addition, the ZIC2 was knocked down by siRNA to further reveal its effects on cell viability, cellular migration and cell cycle of BT549 cells.

### Clinical samples

We obtained 20 pairs of breast cancer tissues and the paired normal adjacent tissues from patients without preoperative chemotherapy, hormone therapy, or radiotherapy who had undergone tumor resection at The First Affiliated Hospital of Fujian Medical University between 2020 and 2023, with written consent from all patients concerned.

### Cell lines and Cell Culture

MCF10A, the normal breast epithelial cell line, was obtained from Procell Life Science & Technology Co., Ltd. (Wuhan, China) and cultured in specific epithelial culture medium (CM-0525, Procell Co., Ltd). Furthermore, the human breast cancer cell line BT549 was purchased from Sailybio Co., Ltd. (Shanghai, China) and cultured in RPMI-1640 medium (meilunbio, China) supplemented with 10% fetal bovine serum (FBS; meilunbio, China) and 100 U/ml penicillin and streptomycin in a humidified atmosphere of 5% CO2 at 37 °C.

### RT-qPCR

Total RNA was extracted from cells using the Total RNA Isolation Kit V2 (Vazyme, China), and Complementary DNA (ctDNA) was synthesized by the HiScript III All-in-one RT SuperMix Perfect for qPCR (Vazyme, China). In addition, RT-qPCR was conducted on an ABI 7500 thermocycler (Applied Biosystems, Foster City, CA, USA) using the SYBR Green PCR Master Mix (Vazyme, China), with appropriate primers were listed in Additional File 2. The relative expression levels of mRNA were reported as fold change compared to levels detected in controls via the ΔΔCt method.

### IHC

By means of the standard avidin-biotin complex method, the staining procedure was performed for IHC, with the extent of positively stained cells scored (0–10). In addition, the ZIC2-stained sections were divided into two groups (High and Low) based on scores of the staining between breast cancer tissues and paired normal adjacent tissues, with two pathologists evaluating all the specimens in a blinded manner.

### SiRNA transfection

For the ZIC2 depletion studies, BT549 cells were plated at a density of 3 × 10^5^ cells/well in 6-well plates. After reaching 30–40% confluence, cells were transfected with siRNA and GP-transfect-Mate (Shanghai GenePharma Co., Ltd.) according to the protocol of manufacturer. The siRNA duplexes with the following sense and antisense sequences were used: GAACCUCAAGAUCCACAAATT and UUUGUGGAUCUUGAGGUUCTT. All of the siRNAs were synthesized by Hanbio (Hanbio Biotechnology Co., Ltd., Shanghai, China), and the cells were transfected with siRNAs via GP-transfect-Mate (Shanghai GenePharma Co., Ltd.) for 48 h and then harvested for further experiments.

### CCK-8 assay

Cells were incubated at 5% CO2 and 37 °C on 96-well plates (100 µL/well), and ten microliters of CCK-8 reagent (Beyotime Bio Inc., China) were added to each well after 24 h, 48 h, 72 h, and 96 h, respectively, with OD450 values determined by a microplate reader.

### Cellular Migration Assay

The cellular migration assay was performed in vitro using 24-well transwell chambers. Cells (2 × 10^^4^) were seeded in the top chambers, and the bottom chambers were filled with RPMI-1640 medium (600 ml) containing 10% FBS to stimulate migration. After 24 h of incubation, the cells were stained with 0.1% crystal violet. The cells that had migrated through the ostioles to the reverse side were counted under a microscope in five predetermined fields at magnification of 200. Each assay was performed in triplicate.

### Cell cycle assay

Cells were harvested and fixed in 70% cold ethanol and stored at 4 °C overnight. The next day cells were centrifuged at 2500 rpm for 5 min. After washed with PBS, cell pellets were treated with RNase A and stained with PI at 37 °C for 30 min in the dark. The cell cycle was assessed with flow cytometry (Beckman Coulter).

### Protein extraction and western blot analysis

The cells were lysed using Radio Immunoprecipitation Assay (RIPA) lysis buffer (Beyotime, China). The protein concentration was determined by BCA protein concentration kit (Beyotime, China). Sodium dodecyl sulfate-polyacrylamide gel electrophoresis (SDS-PAGE) was done in advance to separate the proteins. The proteins were subsequently electroblotted onto polyvinylidene difluoride membranes (Millipore, 150 Billerica, MA, USA). The membranes were blocked for 2 h in a solution of 5% nonfat dry milk in Tris Buffer Solution Tween (TBST). The primary antibodies ZIC2 (ZIC Family Member 2; HUABIO, 1: 2000) and β-actin (#YT0099, 1:5000) were then incubated at 4 °C overnight. The membranes were washed with TBST every 10 min. Membranes were washed and stained for 1 h at room temperature with goat anti-rabbit IgG secondary antibody (HUABIO, 1: 50,000). After that, the membranes were washed three times with TBST every 10 min. The blots were cut prior to hybridisation with antibodies during blotting. The bands were developed using an improved ECL kit (Thermo Scientific, Rockford, USA). GelDoc XR equipment was used to take the images, which were then analyzed using Image Lab Software (Bio-Rad). The original, uncropped Western Blot images of the study were in Additional File 3.

### Statistical analysis

Statistical analyses in this study were conducted by R software (version 3.6.3). The log-rank test was applied for the KM analysis. T test or Wilcoxon-test were used to compare the risk score between different clinical characteristic subgroups and proportion of immune infiltration score, TIICs, immune checkpoints and chemotactic factors between low-risk and high-risk groups. The mean standard deviation was utilized when the data were normally distributed and the independent t-test was used to compare the means of two independent groups. A p-value of < 0.05 was considered statistically significant. Graphpad Prism 8.0 was used to conduct the statistical analyses (GraphPad Software Inc., USA). *P < 0.05, **P < 0.01, *** *P* < 0.001.

## Results

### Identification of SERGs in breast cancer

**Fig. 1:**
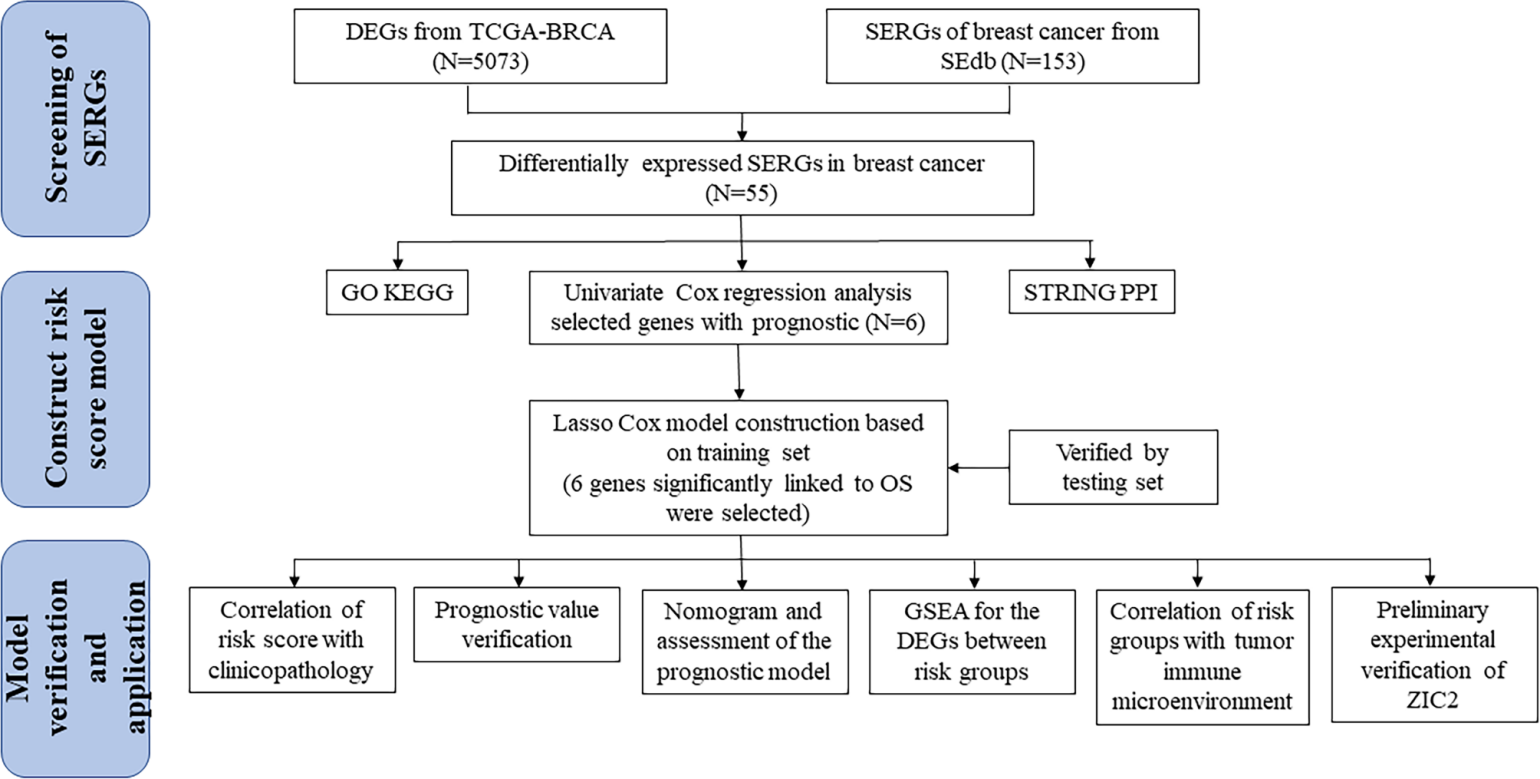
The flow diagram of this study

**Fig. 2:**
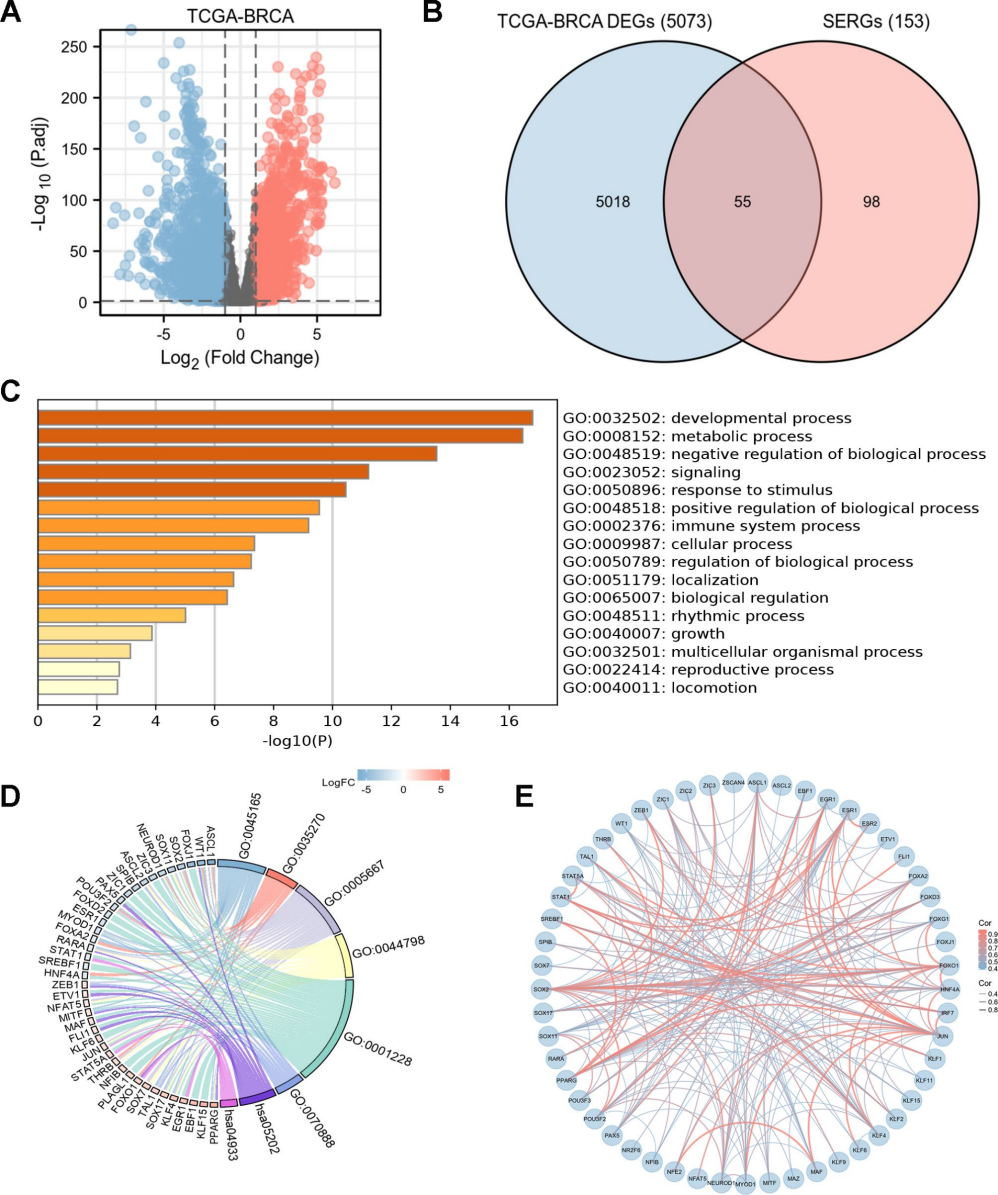
Characterization of differentially expressed SERGs in breast cancer **(A)** Volcano plot of differentially expressed genes (DEGs) in TCGA-BRCA with the cut-offs of |log2 (fold change) | value of > 1 and false discovery rate (FDR) control (adjusted *P* < 0.05); **(B)** 55 SERGs were screened via Venn diagram of DEGs from TCGA-BRCA and SERGs from SEdb; **(C, D)** Gene ontology (GO) and Kyoto Encyclopedia of Genes and Genomes (KEGG) (www.kegg.jp/kegg/kegg1.html) analyses of the 55 differentially expressed SERGs; **(E)** protein-protein interaction (PPI) networks constructed by STRING on the basis of 55 differentially expressed SERGs

The flow of the study was displayed in Fig. 1. After raw data preprocessing, a total of 5,073 DEGs of TCGA-BRCA (Fig. 2A) was obtained (the details of 5073 DEGs and GSEA for them were shown in Additional File 4, GSEA for the whole transcriptome were shown in Additional File 4). Meanwhile, 153 SERGs were identified from SEdb 2.0 after removal of duplicates. Then, based on overlapping genes between the two algorithms mentioned above, a total of 55 SERGs were screened for further analysis (Fig. 2B) (the details of 55 SERGs were shown in Additional File 5).

### Functional clustering analysis of SERGs in breast Cancer

Go and KEGG analyses were performed to reveal the functions of the 55 SERGs. These genes were significantly enriched in terms of developmental process, metabolic process, and tumor formation (Fig. 2C, D). In addition, the protein-protein interaction (PPI) network among these genes illuminated that they interacted with each other (Fig. 2E).

### Construction and validation of the SERGs Prognostic signature

**Fig. 3:**
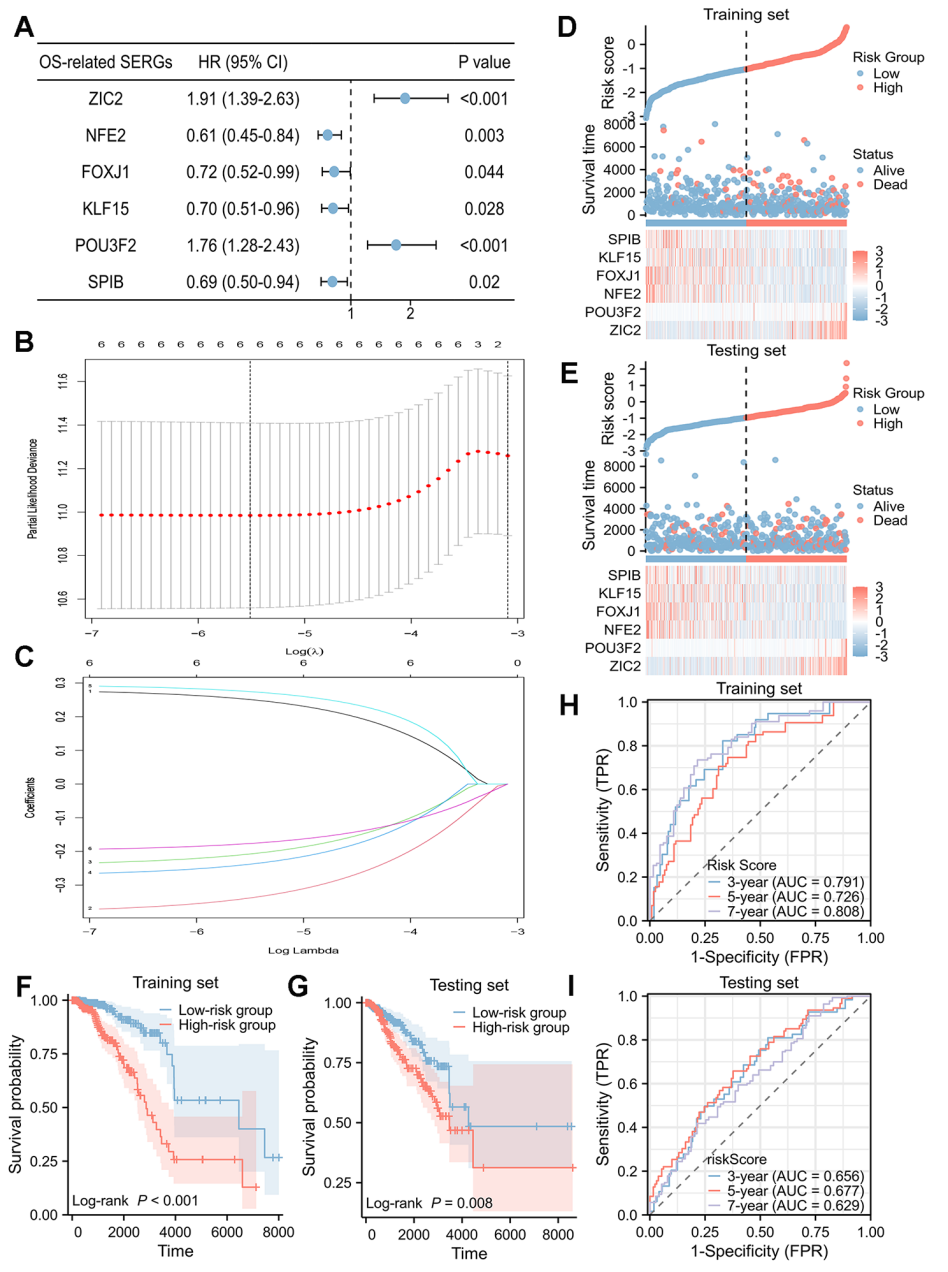
Identification and validation of the 6-SERGs signature **(A)** The 6 differentially expressed SERGs with prognostic value were extracted by univariate Cox regression analysis and represented by a forest plot; **(B)** the tenfold cross-validation for variable selection in the LASSO model; **(C)** the LASSO coefficient profile of 6 differentially expressed SERGs; risk plot distribution, survival status of patients, and heat map including 6 SERGs in **(D)** training set and **(E)** testing set; Kaplan-Meier (KM) survival curves of overall survival (OS) for patients between low-risk and high-risk groups in **(F)** training set and **(G)** testing set; time-dependent receiver operating characteristic (ROC) curves for predictive performance of the SERGs signature in **(H)** training set and **(I)** testing set

Univariate Cox regression analysis was used to screen the SERGs with prognostic value in breast cancer. Then, 6 SERGs were identified as potential prognostic genes (Fig. 3A) by cut-off threshold of *P* < 0.05. Then, LASSO regression algorithm was used to refine by calculating regression coefficients in training set (Fig. 3B, C). Finally, the 6-SERGs prognostic model, comprised of POU3F2, NFE2, FOXJ1, KLF15, SPIB, and ZIC2, were obtained, with the risk score system was set up (Table 1) (the relationships between individual composition gene and clinical outcomes were detailed in Additional File 6).

**Table 1:** Differentially expressed SERGs and their coefficients in LASSO regression model

Gene	Description	Coefficients
ZIC2	Zic Family Member 2	0.251
NFE2	Nuclear Factor Erythroid 2	-0.339
FOXJ1	Forkhead Box J1	-0.208
KLF15	Kruppel-like factor 15	-0.238
POU3F2	POU Class 3 Homeobox 2	0.275
SPIB	Spi-B Transcription Factor	-0.177

SERGs, super-enhancer-related genes; LASSO, Least Absolute Shrinkage and Selection Operator

DNA methyltransferases on CpG island methylation are transcription factors in the suppression or promotion of cell growth which is a reversible process [33]. In this study, we present the heatmap and prognostic value of DNA methylation clustering the expression levels of the 6 SERGs in breast cancer (Additional File 7). With regard to DNA methylation expression levels, cg06807713, cg25721818, cg23384027, cg17869315, cg24087497 from NFE2; cg16861241, cg12284789, cg25051233 from FOXJ1 came up with the highest levels and significant prognostic values (likelihood ratio (LR) test *P* < 0.05) in breast cancer.

With the risk score formula, the distribution of risk score, survival status, survival time, and expression levels of 6 SERGs for breast cancer were compared between low-risk and high-risk groups in both training (Fig. 3D) and testing (Fig. 3E) sets. The results indicated the high-risk group had a worse prognosis. KM analysis displayed that high-risk group had a significantly poorer OS than low-risk group in both training (*P* < 0.001, Fig. 3F) and testing (*P* = 0.008, Fig. 3G) sets. Besides, the time-dependent ROC curve in training (Fig. 3H) and testing (Fig. 3I) sets indicated that the SERGs prognostic signature was reliable for predicting the prognosis of breast cancer patients.

### Risk score and clinicopathological characteristics

**Fig. 4:**
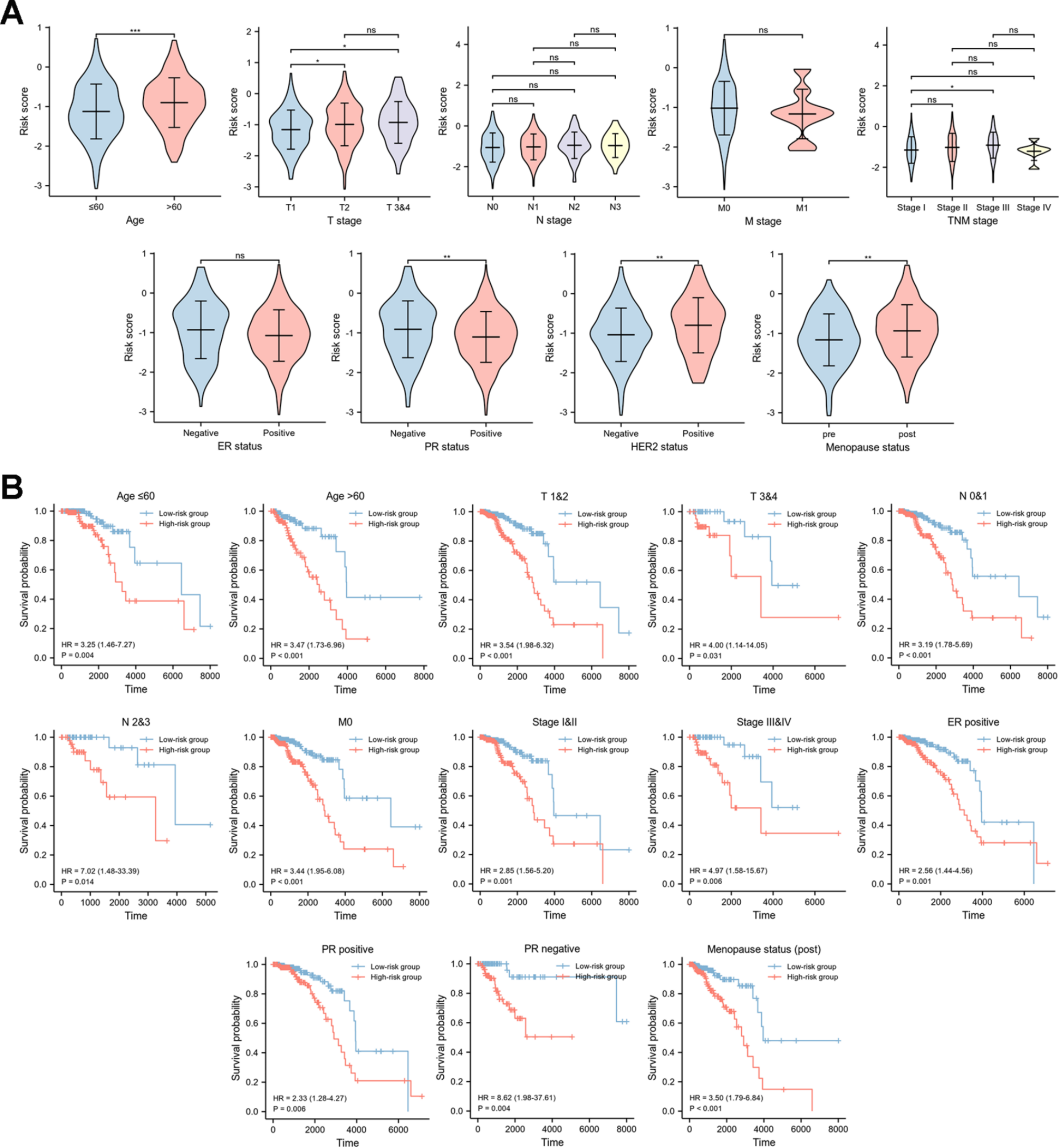
The predictive power of the risk score system **(A)** Correlation between the risk score and clinical characteristics (age; T stage; N stage; M stage; TNM stage; ER status; PR status; HER2 status; menopause status); **(B)** KM curves for OS prediction in subgroups based on various clinical characteristics (age ≤ 60 years; age > 60 years; T1&2; T3&4; N0&1; N2&3; M0; stage I&II; stage III&IV; ER positive; PR positive; PR negative; menopause status (post))

Relationship between risk score and clinicopathological characteristics of patients in training set were analyzed in Fig. 4A. It revealed that risk score was lower in breast cancer patients ≤ 60 years, T1 stage, PR positive, HER2 negative, and pre menopause status. In addition, we also validated the prediction efficiency of risk groups in several subgroups. KM analyses showed that high-risk group had a worse OS in subgroups of age ≤ 60 years, age > 60 years, stages of T1&2, T3&4, N0&1, N2&3, M0, TNM stage I&II, TNM stage III&IV, ER positive, PR positive, PR negative, and post menopause status (Fig. 4B). In addition, subgroup analysis revealed that the high-risk group had a significantly worse OS in luminal A, B breast cancer and tended to have a worse clinical outcome in triple negative breast cancer (Additional File 8).

### Nomogram and Validation

**Fig. 5:**
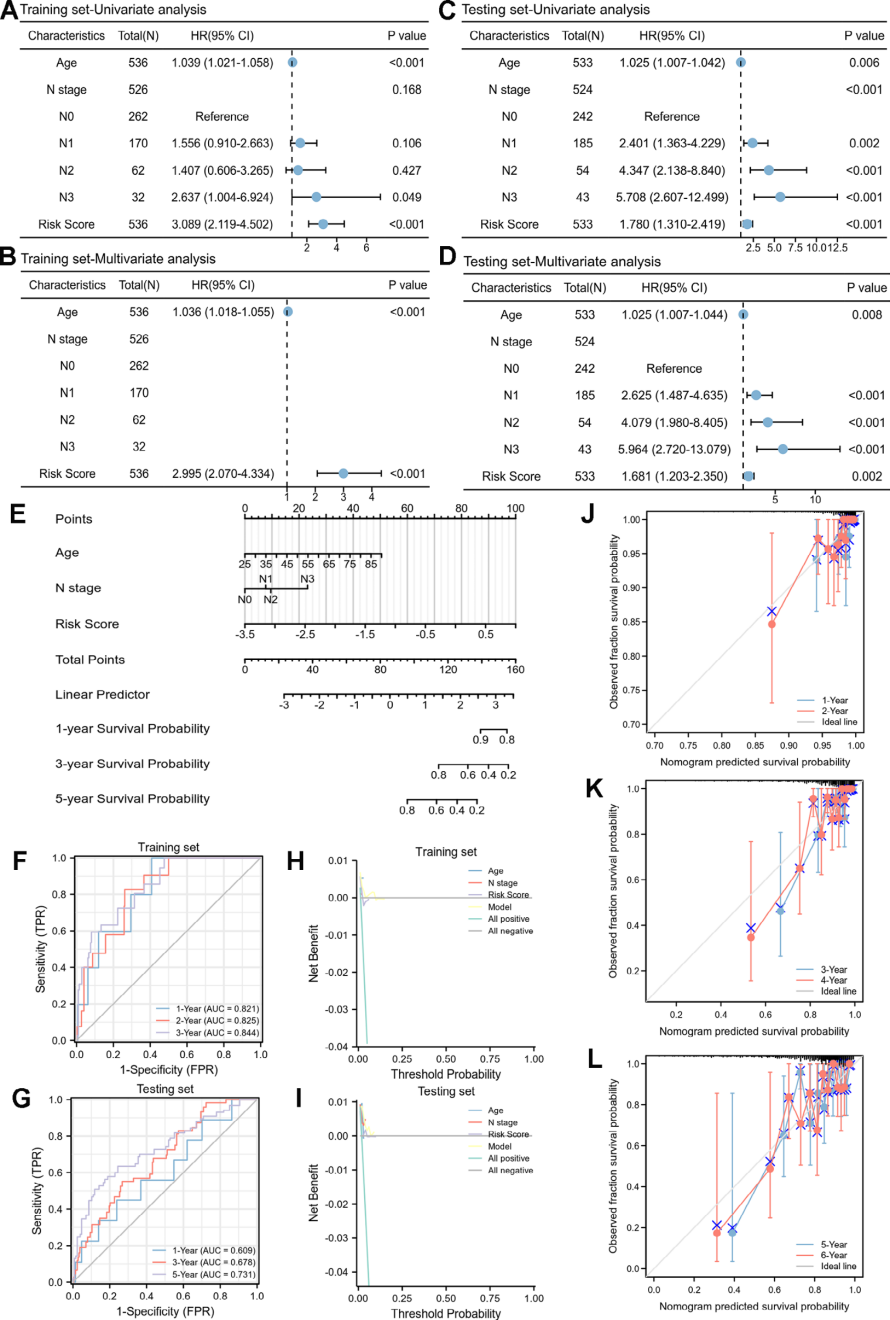
Nomogram and verification of prognostic model Univariate and multivariate Cox analyses of clinical factors and risk score with OS in training set **(A, B)** and testing set **(C, D)**; **(E)** nomogram predicting 1-, 3- and 5-years survival rate of breast cancer patients; time-dependent ROC curves for predictive performance of the model in **(F)** training set and **(G)** testing set; decision curve analysis (DCA) for the model in **(H)** training set and **(I)** testing set; the calibration curves for **(J)** 1- and 2-, **(K)** 3- and 4-, **(L)** 5- and 6-year OS in training set

The potential prognostic clinicopathological factors (age, stage of T, N, M, status of ER, PR, HER2, menopause, and radiotherapy) were analyzed by univariate and multivariate Cox regression in training set (Table 2). Then, the independent prognostic parameters (age and N stage) and risk score were calculated by univariate and multivariate Cox regression (Fig. 5A, B). The result showed that high-risk group had significantly lower OS in training set [hazard ratio (HR) = 2.995, 95% confidence interval (CI): 2.070–4.334, *P* < 0.001]. The model was verified by testing set (HR = 1.681, 95% CI: 1.203–2.350, *P* = 0.002) (Fig. 5C, D). Subsequently, the age, N stage and risk score were integrated into nomogram model (Fig. 5E) with the C-index was 0.788 (95% CI: 0.759–0.816). Moreover, the results of time-dependent ROC analysis (Fig. 5F, G) and DCA (Fig. 5H, I) for model in training and testing sets revealed that the discriminatory ability of the nomogram was robust. In addition, the calibration plots displayed outstanding agreement among 1- and 2-, 3- and 4-, 5- and 6-year OS rates when comparing the ideal and nomogram model (Fig. 5J, K, L).

**Table 2:** Univariate and multivariate Cox analysis of OS in training set

Characteristics	Total (N)	Univariate analysis			Multivariate analysis	
		HR (95% CI)	*P* value		HR (95% CI)	*P* value
Age	536	1.039 (1.021–1.058)	**< 0.001**		1.054 (1.026–1.083)	**< 0.001**
T stage	534					
T1	139	Reference				
T2	316	1.168 (0.674–2.025)	0.580			
T3&4	79	1.119 (0.539–2.324)	0.762			
N stage	526					
N0	262	Reference				
N1	170	1.556 (0.910–2.663)	0.106			
N2	62	1.407 (0.606–3.265)	0.427			
N3	32	2.637 (1.004–6.924)	**0.049**			
M stage	451					
M0	442	Reference				
M1	9	1.455 (0.567–3.733)	0.435			
ER status	506					
Negative	110	Reference				
Positive	396	1.316 (0.681–2.543)	0.413			
PR status	505					
Negative	162	Reference				
Positive	343	1.030 (0.601–1.766)	0.914			
HER2 status	338					
Negative	267	Reference				
Positive	71	1.912 (0.912–4.011)	0.086			
Menopause status	459					
Pre	117	Reference			Reference	
Post	342	2.605 (1.167–5.811)	**0.019**		0.737 (0.256–2.123)	0.571
Radiotherapy	91					
YES	42	Reference				
NO	49	1.274 (0.283–5.745)	0.753			

OS, overall survival; ER, estrogen receptor; PR, progesterone receptor; HER2, human epidermal growth factor receptor 2; HR, hazard ratio; 95% CI, 95% confidence interval

### DEGs between high-risk and low-risk groups

**Fig. 6:**
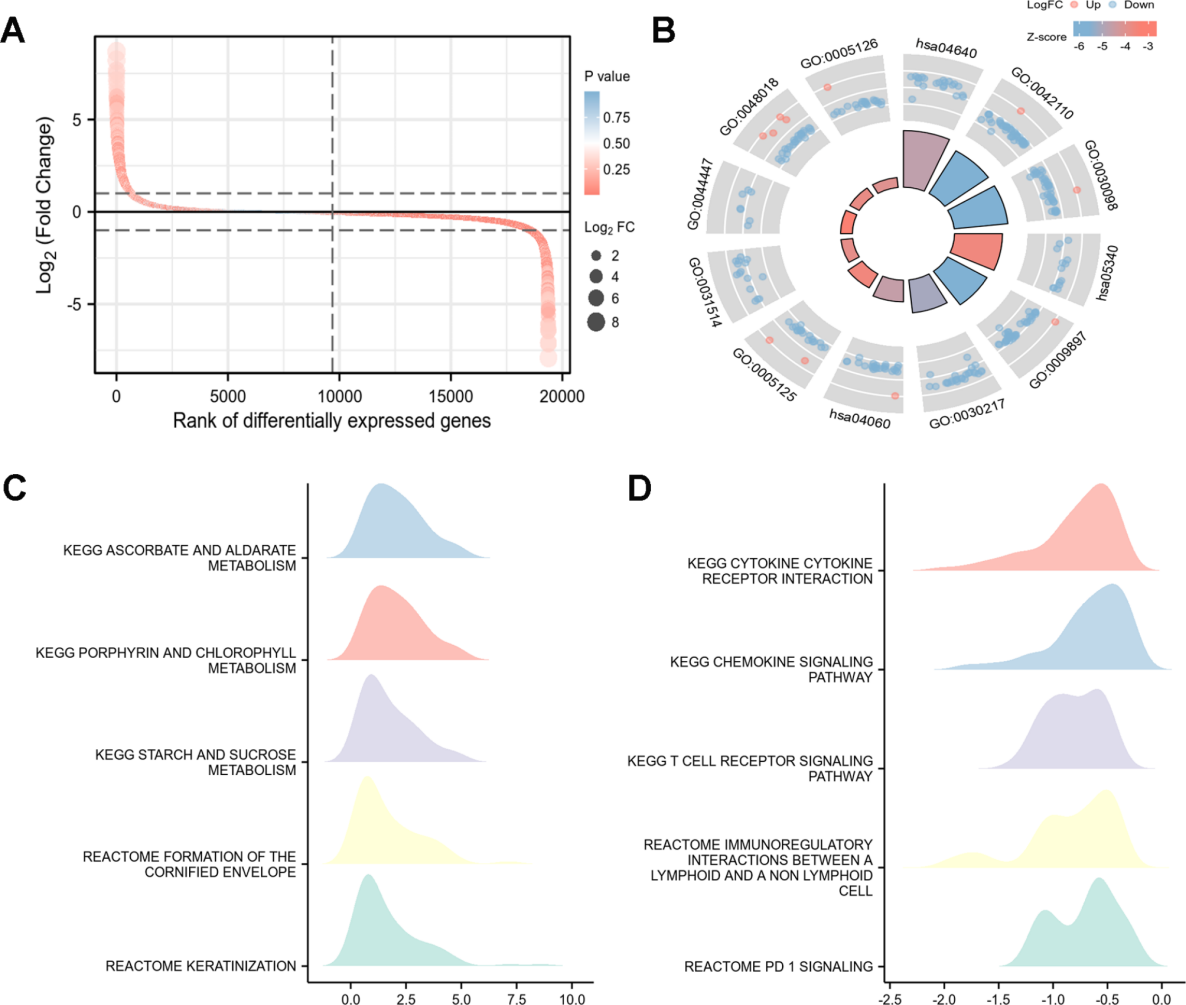
The DEGs between low-risk and high-risk groups **(A)** Variance ranking chart and **(B)** GO and KEGG (www.kegg.jp/kegg/kegg1.html) analyses of DEGs from high-risk group compared with low-risk group; **(C, D) **ridge map of gene set enrichment analysis (GSEA) for DEGs from high-risk group compared with low-risk group

Based on the DEGs between low-risk and high-risk groups (Fig. 6A), Go and KEGG analyses were performed and showed these genes were significantly enriched in terms of T cell activation, lymphocyte differentiation, receptor ligand activity, and cytokine-cytokine receptor interaction (Fig. 6B). Moreover, GSEA analysis was conducted to clarify the potential regulatory mechanisms leading to the differences between low-risk and high-risk groups. Some cancer-related hallmarks, including the cancer metabolism and dysregulation of cell cycle were significantly associated with high-risk group (Fig. 6C). T cell receptor signaling pathway, chemokine signaling pathway and PD-1 signaling pathways were associated with low-risk group (Fig. 6D).

### Associations between SERGs signature and TIME

**Fig. 7:**
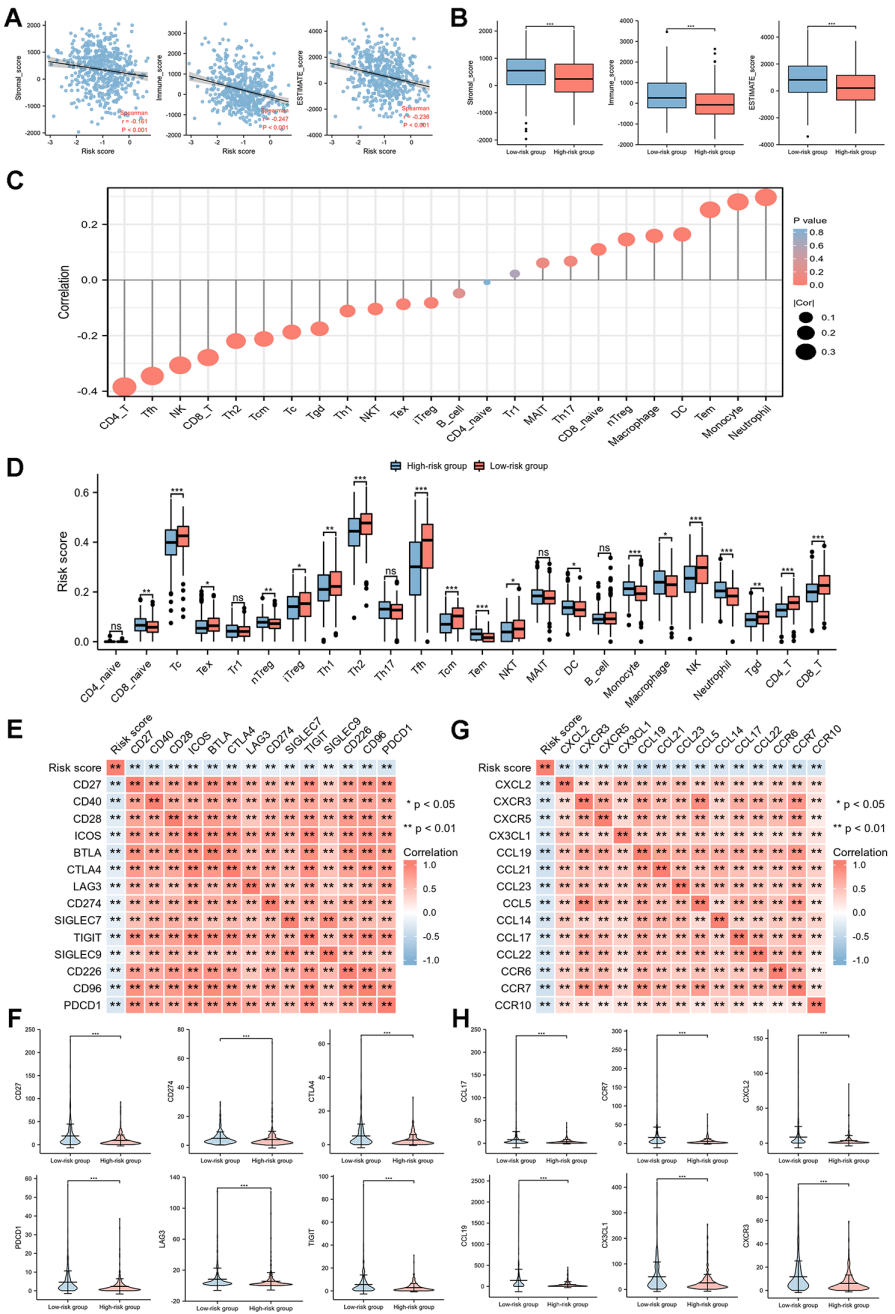
Correlation between prognostic model and TIME **(A)** Relationship between immune infiltration scores (including Stromal score, Immune score, and ESTIMATE score) and risk score; **(B)** comparison of immune infiltration scores between low-risk and high-risk groups; **(C)** correlations between the risk model and tumor-infiltrating immune cells (TIICs); **(D)** comparisons of TIICs between low-risk and high-risk groups; **(E)** association between risk score and immune checkpoints; **(F)** comparison of six immune checkpoints between low-risk and high-risk groups; **(G)** association between the risk score and chemotactic factors; **(H)** comparison of six chemotactic factors between low-risk and high-risk groups

The results displayed that the risk score was negatively correlated with immune infiltration score (Stromal score, Immune score and ESTIMATE score) (Fig. 7A, B); furthermore, the risk score was negatively correlated with tumor-infiltration lymphocytes (TILs) (such as CD4 + T cells, NK, and CD8 + T cells) (Fig. 7C, D). As analyzed with the TIMER tool, expressions of SERGs were also correlated with immune infiltration profiles in breast cancer. In summary, expression of each SERG was associated with tumor purity and markers of different immune cells (Additional File 9).

Moreover, we evaluated the relationship between risk score and immune checkpoints (Fig. 7E). The expression level of immune checkpoints (including CD27, CD274, CTLA4, PDCD1, LAG3, TIGIT) were lower in high-risk group (Fig. 7F).

Besides, we also analyzed the relationship between risk score and chemotactic factors (Fig. 7G). The expression level of chemotactic factors (including CCL17, CCR7, CXCL2, CCL19, CX3CL1, CXCR3) were lower in high-risk group (Fig. 7H).

### Preliminary Experimental Verification for ZIC2 Gene

**Fig. 8:**
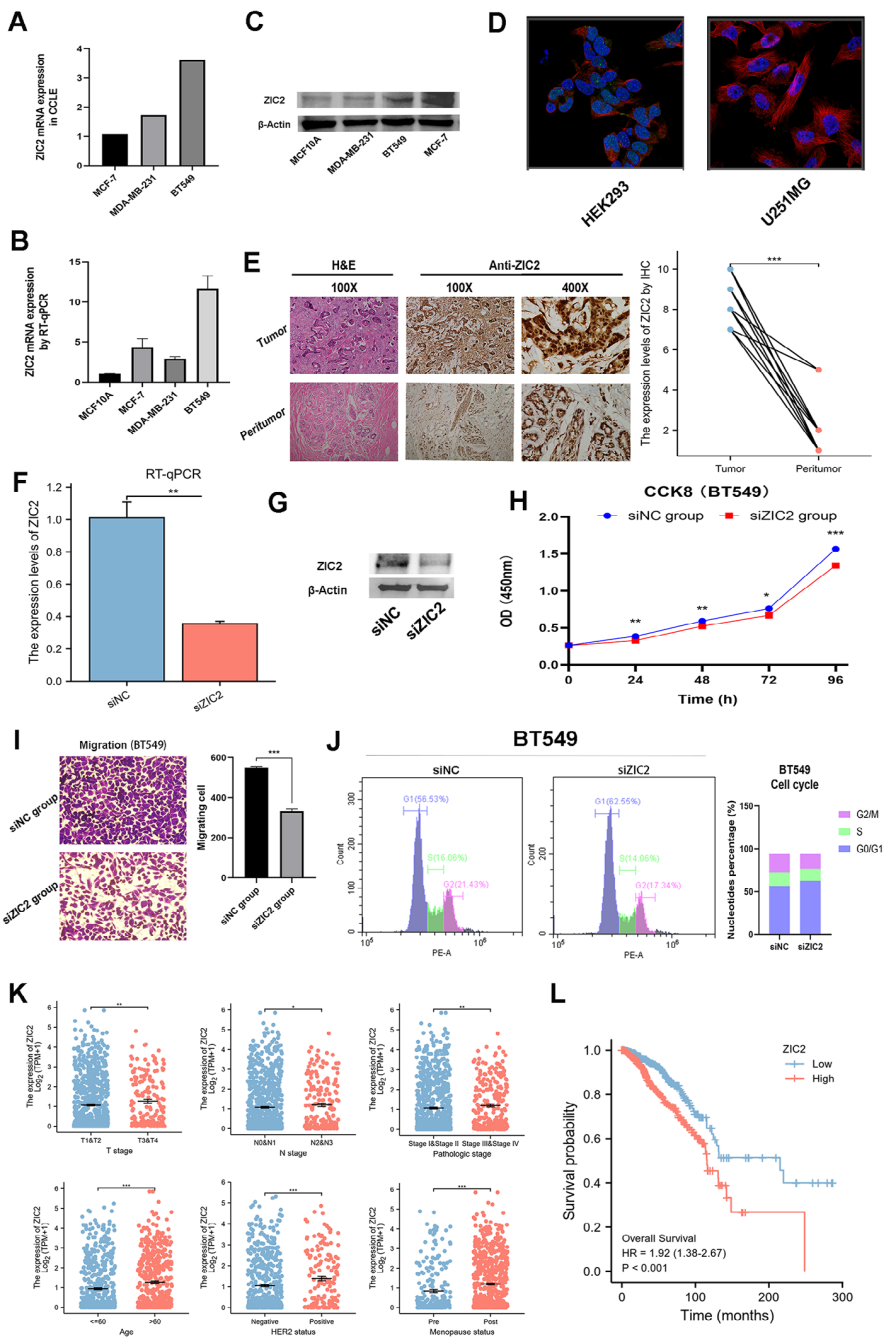
Preliminary experimental verification of characteristics of ZIC2 **(A)** ZIC2 mRNA expression levels in breast cancer cell lines from CCLE; **(B)** the mRNA relative expression levels of ZIC2 in a normal breast cell line (MCF10A) and breast cancer cell lines (MCF-7, MDA-MB-231 and BT549) determined by RT-qPCR. **(C)** the protein expression levels of ZIC2 in a normal breast cell line (MCF10A) and breast cancer cell lines (MCF-7, MDA-MB-231 and BT549) determined by cropped Western Blot; **(D)** ICC for determining the subcellular location of ZIC2 in HEK293 and U251MG cell lines by HPA. ZIC2 localized to the nucleoplasm and nuclear bodies (green). Microtubules are stained in red and the nucleus in blue (DAPI); **(E)** the representative images of H&E and IHC for ZIC2 expressed on breast cancer and peritumoral tissues, and the protein expression levels of ZIC2 in 20 pairs of tumoral and peritumoral tissues by IHC; the mRNA and protein expression levels of ZIC2 in BT549 cells treated with siNC or siZIC2 via **(F)** RT-qPCR and **(G)** cropped Western Blot; **(H)** the cell viability of BT549 cells treated with siNC or siZIC2 by CCK-8 assay; **(I)** cellular migration assay of BT549 cells treated with siNC or siZIC2; **(J)** cell cycle assay of BT549 cells treated with siNC or siZIC2; **(K)** the association between ZIC2 expression levels and clinicopathological features in breast cancer; **(L)** KM survival curves of OS for breast cancer patients according to the expression level of ZIC2. *P < 0.05, **P < 0.01, ***P < 0.001

We focused on the expression, clinical significance and prognostic value of ZIC2 in breast cancer to further confirm the prognostic signature. ZIC2 expression at the transcriptome level in breast cancer cell lines was displayed using the database of CCLE (Fig. 8A) and RT-qPCR (Fig. 8B). Then, ZIC2 expression was further confirmed at the protein level in cell lines using Western Blot (Fig. 8C). These findings showed that ZIC2 was highly expressed in MCF-7, MDA-MB-231, and BT549 cell lines, in accordance with the transcriptional results. The HPA database was used to reveal the subcellular localization of ZIC2 protein in HEK293 and U251MG cells by ICC, which indicated that ZIC2 was primarily expressed in the nucleoplasm and nuclear bodies (Fig. 8D). To confirm the dysregulation of ZIC2, IHC analysis was also performed on 20 pairs of breast cancer and peritumoral tissues (Fig. 8E). Furthermore, we knocked down the ZIC2 by siRNA (Fig. 8F, G). Then, the results of CCK-8 assay (Fig. 8H), cellular migration assay (Fig. 8I) and cell cycle assay (Fig. 8J) showed that the down-regulation of ZIC2 gene expression inhibited the cell viability, cellular migration, and cell cycle of breast cancer cells. In addition, the expression level of ZIC2 was higher in T3&4 stage, N2&3 stage, TNM stage III&IV, age > 60 years, HER2 positive, and post menopause status (Fig. 8K). Patients with high expression level of ZIC2 had a shorter OS (HR = 1.92, 95% CI: 1.38–2.67, *P* < 0.001) (Fig. 8L).

## Discussion

SEs are a subclass of enhancers that frequently contain multiple enhancer-like elements, characterized by dense binding of master transcription factors (TFs) and Mediator complexes and high signals of active histone marks [12, 18]. More and more studies have revealed that as important regulatory regions, SEs control cell identity and contribute to the pathogenesis of diverse diseases [19, 34, 35]. In cancer, SEs have multifaceted roles by activating various oncogenes and other cancer-related genes and play important regulatory roles in cancer occurrence, cell differentiation, immune response and other important biological processes [13, 36].

Recently, the role of SEs in the pathogenesis and prediction of breast cancer has been the research hot spots [37, 38]. However, the underlying mechanism remains to be fully elaborated. Therefore, we performed a comprehensive profiling based on SERGs in breast cancer patients to investigate and establish a prognostic model. In addition, we also explored the relationship between the prognostic signature and TIME.

In this study, we identified a total of 5,073 DEGs from TCGA-BRCA and 153 SERGs from SEdb 2.0. Then, based on overlapping genes between the two algorithms mentioned above, a total of 55 SERGs were screened and established a 6-SERGs signature with prognostic value on the basis of univariate Cox regression analysis and LASSO regression algorithm. Meanwhile, we also validated the prediction efficiency of risk score system in several subgroups. According to the risk score system, breast cancer patients were divided into low-risk and high-risk groups. The effective and stable of the risk score system were validated in training set (including several subgroups) and testing set by KM curve. Then, the age, N stage and risk score were integrated into a nomogram model and calibration plots revealed the robust predictive ability of the prognostic nomogram for OS in the training set. Besides, time-dependent ROC analysis and DCA for model in training and testing sets revealed the well discriminatory ability of the nomogram. Therefore, our prognostic model based on SERGs can competently predict the clinical outcomes of breast cancer patients.

The 6-SERGs prognostic model was comprised of POU3F2, NFE2, FOXJ1, KLF15, SPIB, and ZIC2. Previous studies have indicated that the 6 SERGs are involved in invasion, angiogenesis, and malignant phenotypes of breast cancer [39–44]. Literature evidences have revealed that down-regulation of POU3F2 could remarkably inhibit the increasing trends of proliferation, clone formation, invasion, and migration abilities induced by BCYRN1 in hepatocellular carcinoma (HCC) cells [45]; transcription factor NFE2-related factor 2 (NRF2) could activate antioxidant programs and influence tumorigenesis and metastasis [46]; FOXJ1 could promote bladder cancer cell growth and regulates warburg effect [47]; KLF15 could inhibit the proliferation and migration of gastric cancer cells via regulating the TFAP2A-AS1/NISCH axis [48]; SPIB acted as a tumor suppressor by activating the NFkB and JNK signaling pathways through MAP4K1 in colorectal cancer cells [49]. ZIC2 was overexpressed and played an oncogene role in various cancers, such as lung adenocarcinoma, colorectal cancer, and HCC [50–53]. There is currently limited evidence on the role of ZIC2 in breast cancer. Liu et al. reported that ZIC2 is downregulated and represses tumor growth in breast cancer [54]. However, Zhang et al. argued that ZIC2 was an oncogene and upregulated in breast cancer [44].

In our study, the expression level of ZIC2 in the TCGA databases were negative correlated with OS of breast cancer patients. The mRNA and protein expression levels of breast cancer cell lines were determined, showing that ZIC2 may act as an independent prognostic factor in breast cancer. The in vitro experiments revealed that the down-regulation of ZIC2 gene expression inhibited the cell viability, cellular migration, and cell cycle of breast cancer cells, and ZIC2 might serve as a therapeutic target for breast cancer. In addition, further experimental studies should be conducted to unravel the mechanism by which ZIC2 mediates the malignant function of breast cancer cells, resulting in a poor prognosis for patients.

Then, we performed GO and KEGG analyses based on DEGs between low-risk and high-risk groups to evaluate risk score system. The results displayed that DEGs were significantly enriched in terms of T cell activation, lymphocyte differentiation, receptor ligand activity, and cytokine-cytokine receptor interaction, which were closely related to the tumorigenesis and immune microenvironment [55–58]. Subsequently, we conducted GSEA between high- and low-risk groups. The results revealed that pathways associated with cancer metabolism and dysregulation of cell cycle were enriched in the high-risk group while T cell receptor signaling, chemokine signaling and PD-1 signaling pathways were enriched in the low-risk group. Several studies have showed that TIME was associated with the oncogenesis and prognosis of breast cancer [59–61]. Hence, immune analyses were adopted to further evaluate the relationship between 6-SERGs prognostic model and TIME.

Patients in the two risk groups had different TIME. The results revealed that the risk score was negatively correlated with ESTIMATE score, which was composed of stromal score and immune score. In tumor infiltrating immune cells, the risk score was negatively correlated with tumor-infiltration lymphocytes (TILs) (including CD4 + T cells, NK, and CD8 + T cells), which played an important role in immunotherapy [62–64]. Additionally, the risk score was negatively correlated with both immune checkpoints and chemotactic factors. Those results revealed that tumors of high-risk groups are so-called immunologically “cold”, with immunosuppressive tumor microenvironment [65].

This study was innovative to some extent: it took the SEs as the entry point, with the purpose of discovering potential new biomarker for predicting the prognosis of breast cancer, and a prognostic model on the basis of SERGs was constructed; in addition to validating the model through testing set, the biological function of ZIC2 was also preliminarily explored in vitro experiments. Meanwhile, this study also had three limitations: firstly, it will be better if the performance of the model itself can be further improved; secondly, only one representative risk gene (ZIC2) in the prognostic model was validated in true bench work; thirdly, some patients from TCGA-BRCA lacked of HER-2 status, which might be a cause to the bias.

In summary, the 6-SERGs signature is capable of attributing to screen the population with various TIME and is promising to act as prognostic biomarker for breast cancer patients. Therefore, how to accurately identify patients in high-risk and achieve superior immuno-infiltration of “cold” tumors may be the topic of future research [66–69].

## Conclusions

The novel SERGs signature could predict the prognosis of breast cancer; moreover, SERGs might have a potential role in serving as therapeutic targets for breast cancer.

### Electronic supplementary material

Below is the link to the electronic supplementary material.

Supplementary Material 1

Supplementary Material 2

Supplementary Material 3

Supplementary Material 4

Supplementary Material 5

Supplementary Material 6

Supplementary Material 7

Supplementary Material 8

Supplementary Material 9

## Data Availability

The datasets used and/or analyzed in this study are available from the cancer genome database (TCGA-BRCA) (https://portal.gdc.cancer.gov), SEdb 2.0 (www.licpathway.net/sedb/) (Sample_02_0667; Sample_02_0670; Sample_02_0671; Sample_02_1517), Human Protein Atlas (HPA) database (http://www.proteinatlas.org/), KEGG pathway database (www.kegg.jp/kegg/kegg1.html), Metascape database (https://metascape.org/gp/#/main/step1), STRING (http://string-db.org) (version 11.5), MethSurv database (https://biit.cs.ut.ee/methsurv/), ImmuCellAI database (http://bioinfo.life.hust.edu.cn/ImmuCellAI), GSEA (http://software.broadinstitute.org/gsea/index.jsp), “ggplot2” package (https://ggplot2.tidyverse.org), TIMER (http://timer.cistrome.org/), Cancer Cell Line Encyclopedia (CCLE) (https://sites.broadinstitute.org/ccle).

## Abbreviations

SE: super-enhancer
SERGs: super-enhancer-related genes
DEGs: differentially expressed genes
GO: Gene ontology
KEGG: Kyoto Encyclopedia of Genes and Genomes
KM: Kaplan-Meier
GSEA: gene set enrichment analysis
TCGA: The Cancer Genome Atlas
SEdb: Super-Enhancer database
LASSO: Least Absolute Shrinkage and Selection Operator
OS: overall survival
ROC: receiver operating characteristic
HPA: Human Protein Atlas
C-index: Concordance index
ER: estrogen receptor
PR: progesterone receptor
HER2: human epidermal growth factor receptor 2
HR: hazard ratio
CIs: confidence intervals
TIME: tumor immune microenvironment
ROC: receiver operating characteristic
DCA: decision curve analysis
PPI: protein-protein interaction
STRING: Search Tool for the Retrieval of Interacting Genes
TILs: tumor-infiltration lymphocytes
TIICs: tumor-infiltrating immune cells
IHC: immunohistochemistry
CCK-8: Cell Counting Kit-8
ctDNA: circulating tumor cell DNA
CEA: carcinoembryonic antigen
CA15-3: carbohydrate antigen 15−3
EV: extracellular vesicles
RT-qPCR: Reverse Transcription Quantitative Polymerase Chain Reaction
FDR: false discovery rate
ICC: immunocytochemistry
HCC: hepatocellular carcinoma
