# Supplementary material for: A novel super-enhancer-related gene signature predicts prognosis and immune microenvironment for breast cancer
Source: BMC Cancer. 2023 Aug 18;23:776. doi: 10.1186/s12885-023-11241-2 (PMC10439574; doi:10.1186/s12885-023-11241-2)

**The original, uncropped Western Blot image of the Figure 8C-ZIC2.**


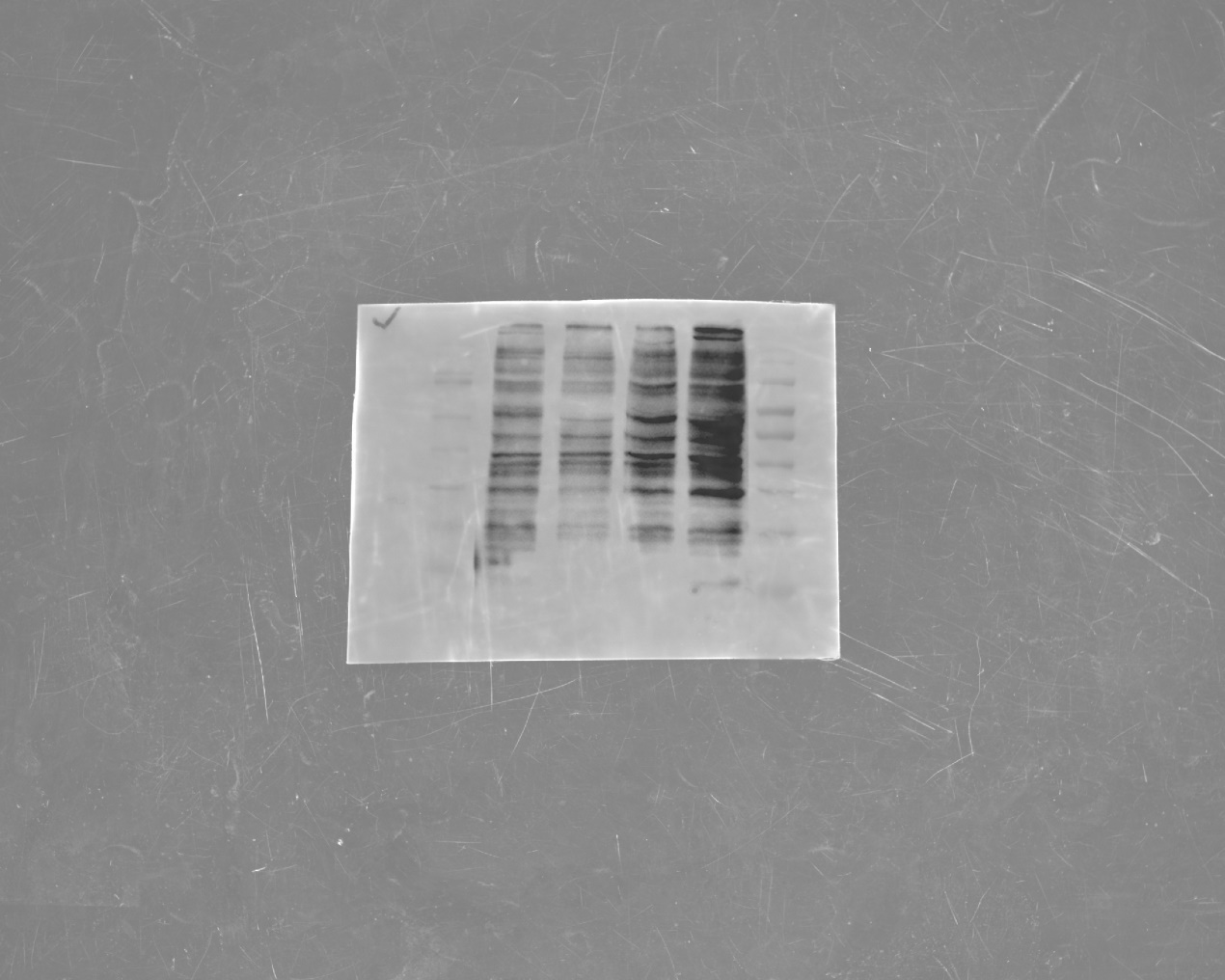


**The original, uncropped Western Blot image of the Figure 8C-Actin.**


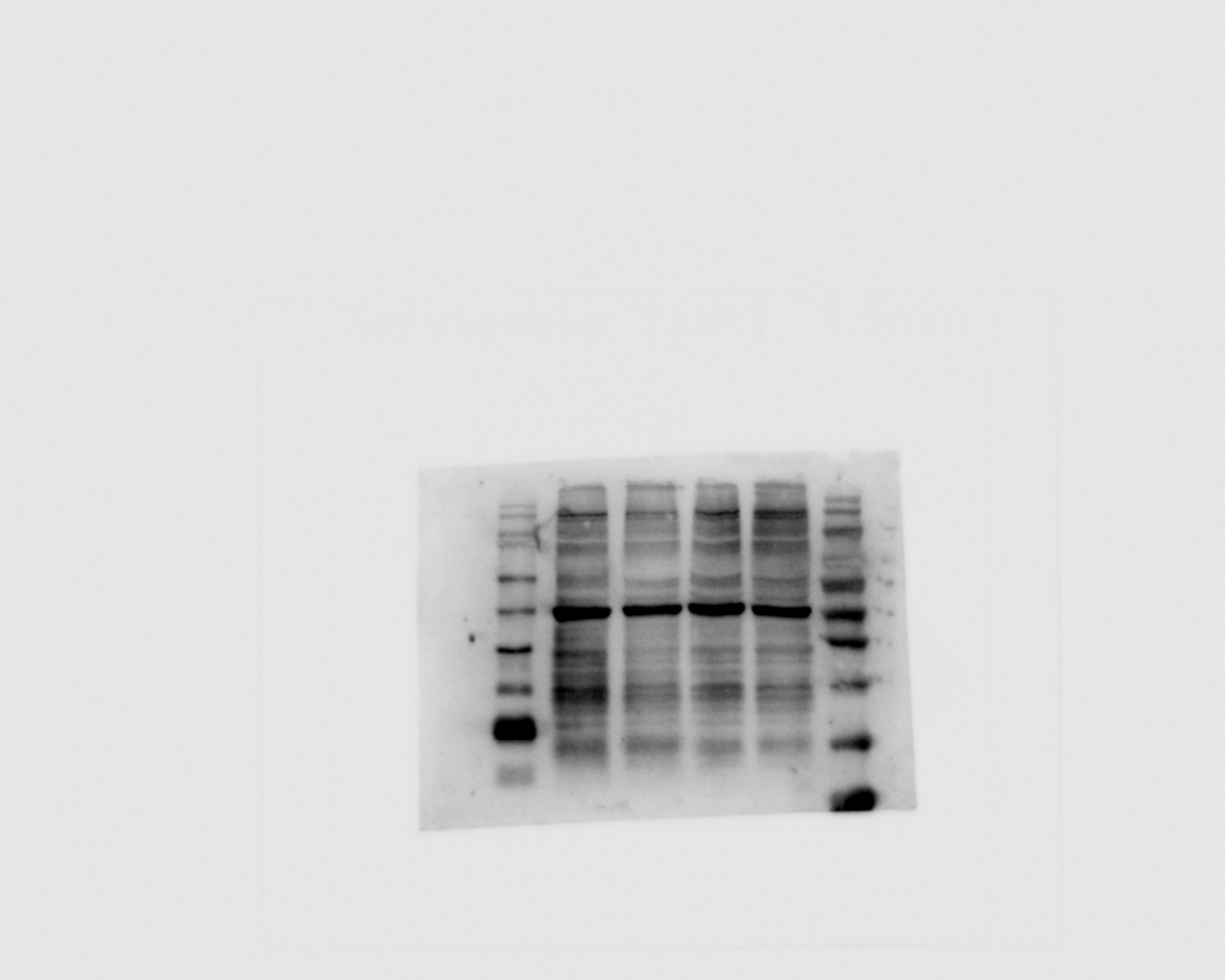


**The photo of the membrane for Figure 8C.**


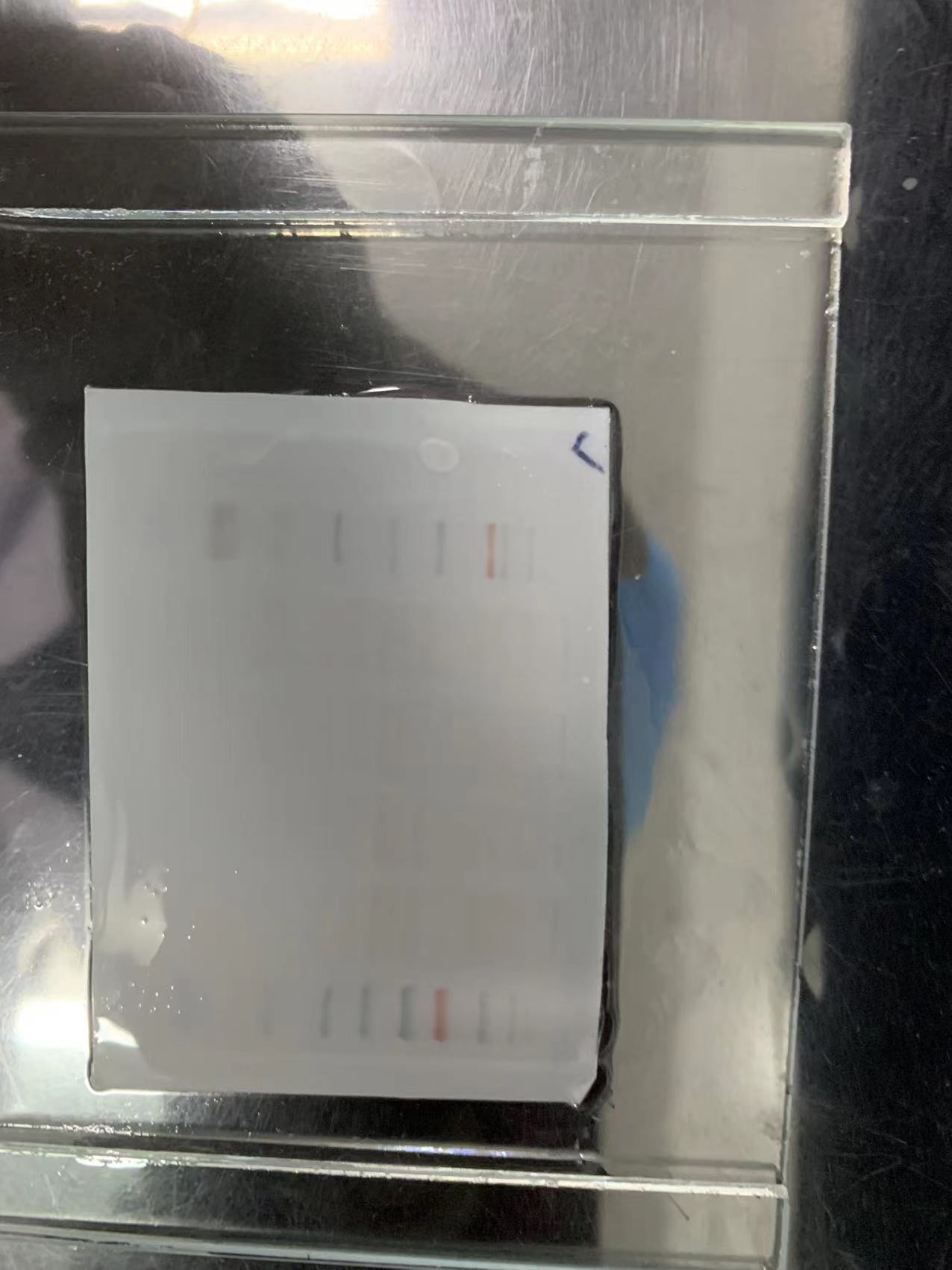


**The original, uncropped Western Blot image of the Figure 8G-ZIC2. (The image in the up was the original, uncropped Western Blot image, the down was the image that has been adjusted for brightness, uncropped Western Blot image)**


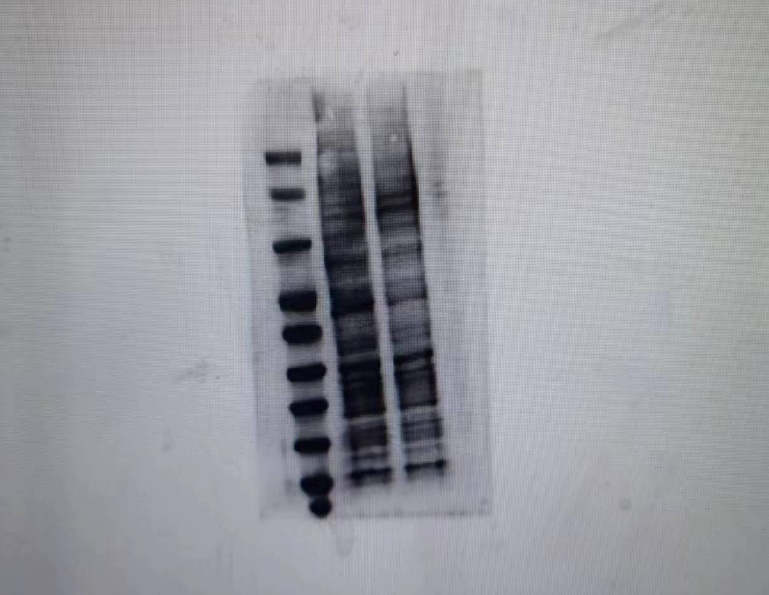


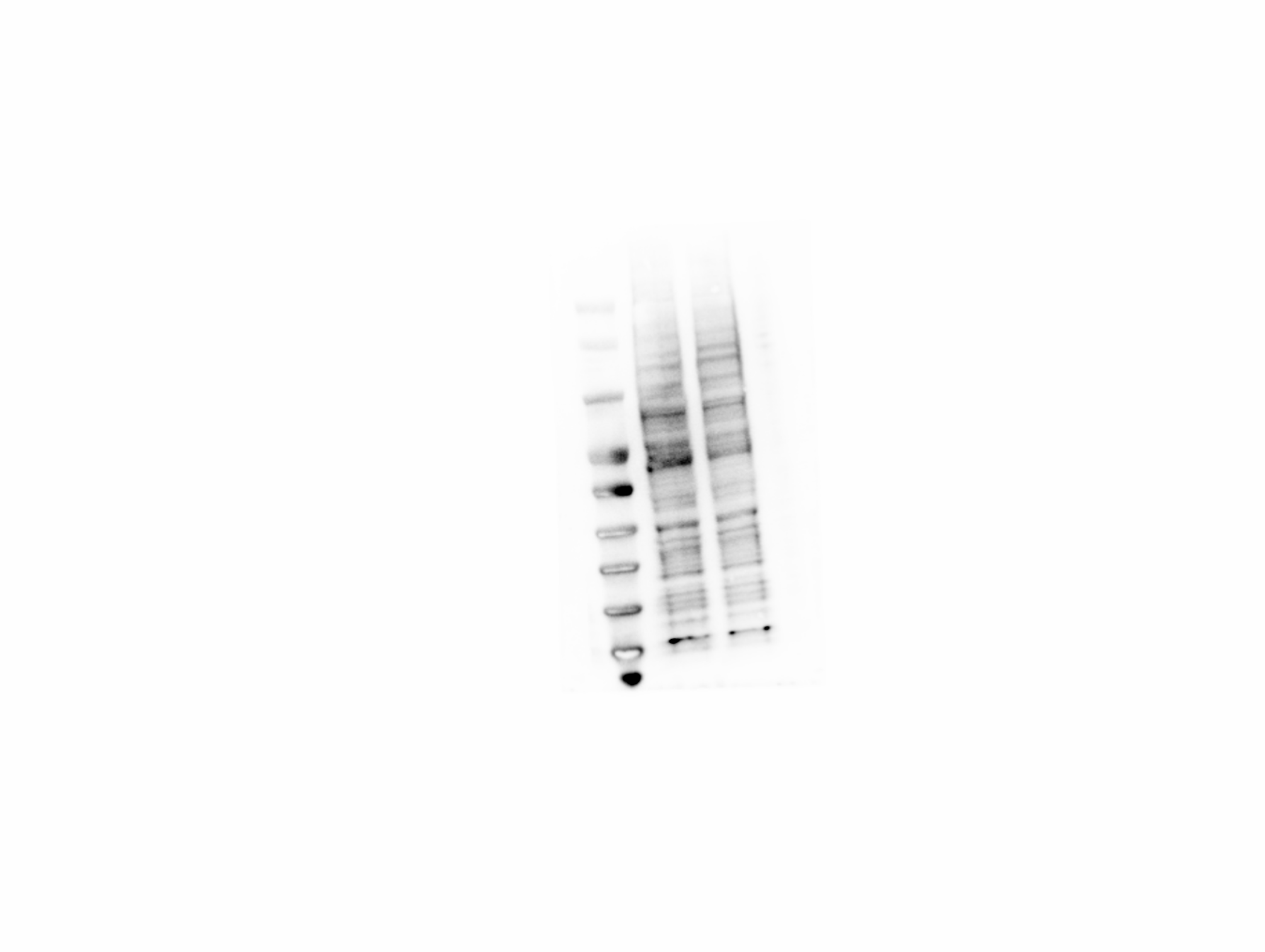


**The original, uncropped Western Blot image of the Figure 8G-Actin.**


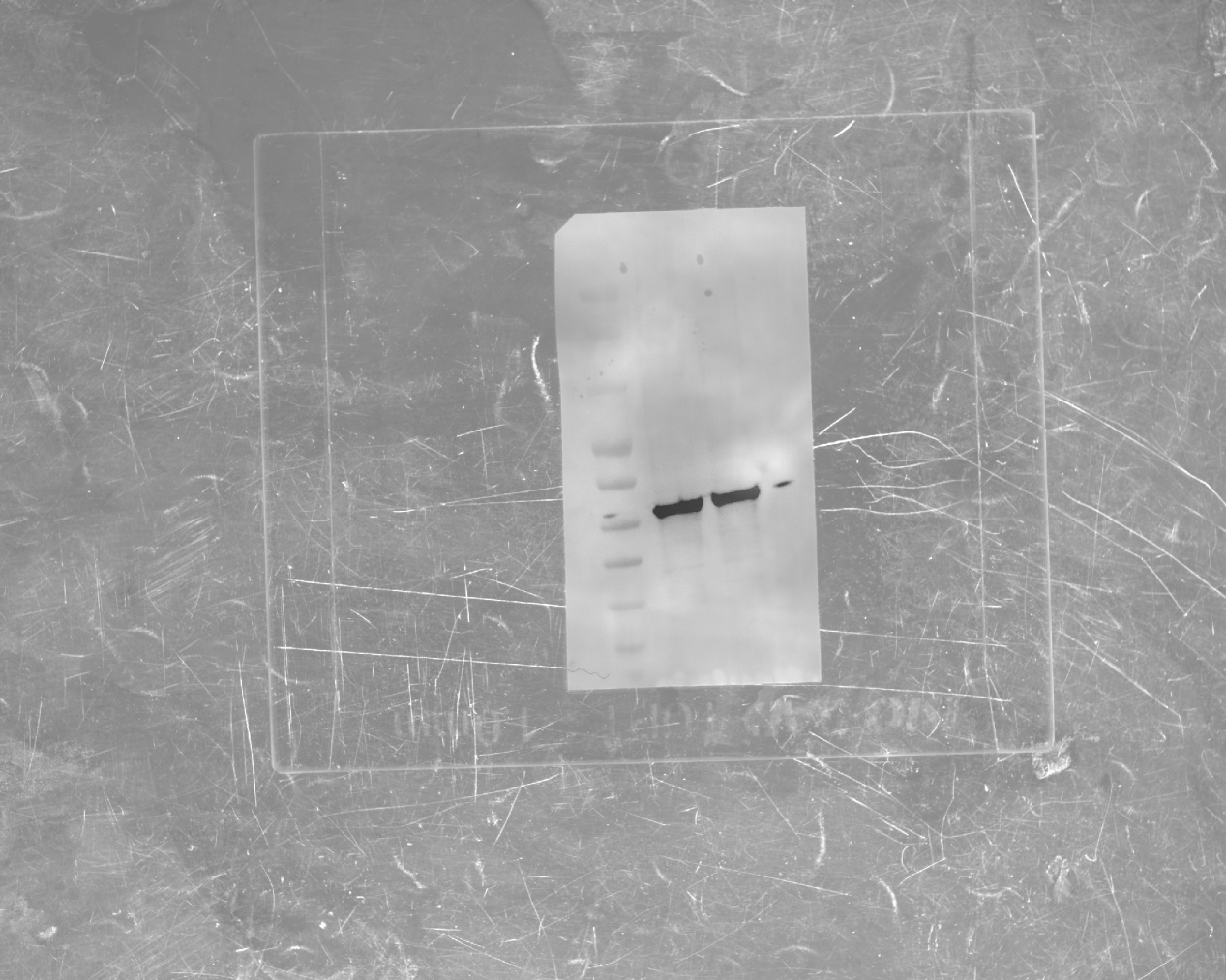


**The photo of the membrane for Figure 8G.**


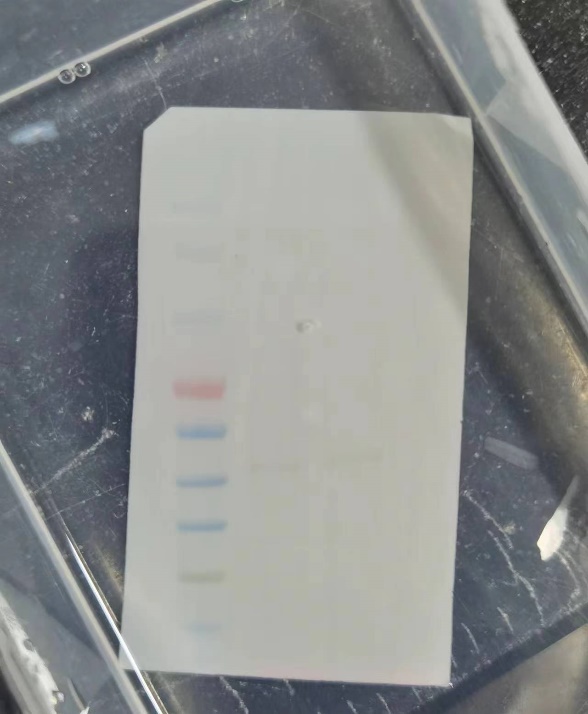

Supplement: Supplementary file 3 — Supplementary Material 3 [file 12885_2023_11241_MOESM3_ESM.docx]
