# Supplementary figures and images for: A novel super-enhancer-related gene signature predicts prognosis and immune microenvironment for breast cancer
Source: BMC Cancer. 2023 Aug 18;23:776. doi: 10.1186/s12885-023-11241-2 (PMC10439574; doi:10.1186/s12885-023-11241-2)

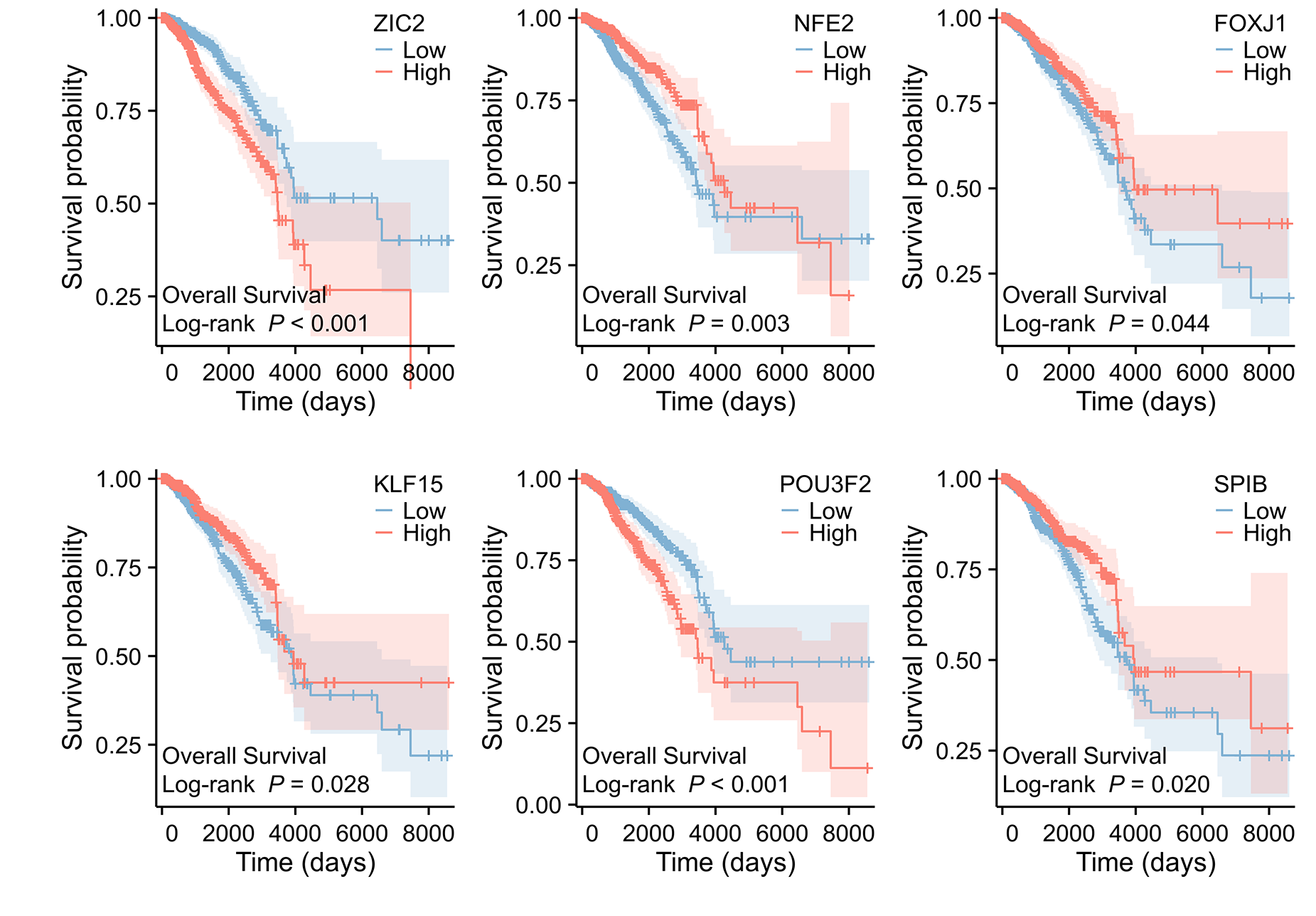

Supplement: Supplementary file 5 — Supplementary Material 5 [file 12885_2023_11241_MOESM5_ESM.tif]

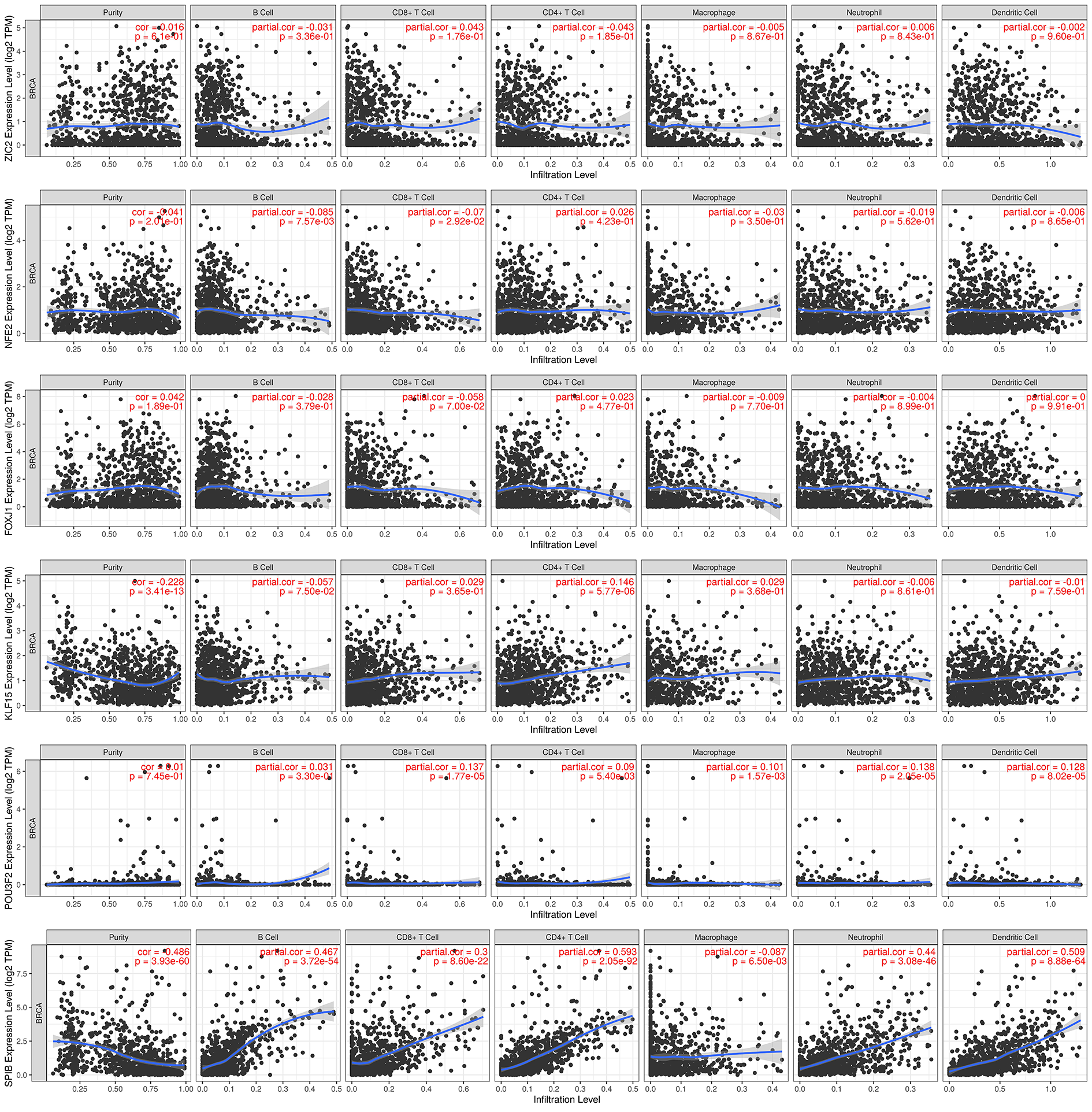

Supplement: Supplementary file 8 — Supplementary Material 8 [file 12885_2023_11241_MOESM8_ESM.tif]
