# Supplementary material for: A novel super-enhancer-related gene signature predicts prognosis and immune microenvironment for breast cancer
Source: BMC Cancer. 2023 Aug 18;23:776. doi: 10.1186/s12885-023-11241-2 (PMC10439574; doi:10.1186/s12885-023-11241-2)
Supplement: Supplementary file 6 — Supplementary Material 6 [file 12885_2023_11241_MOESM6_ESM.docx]

**
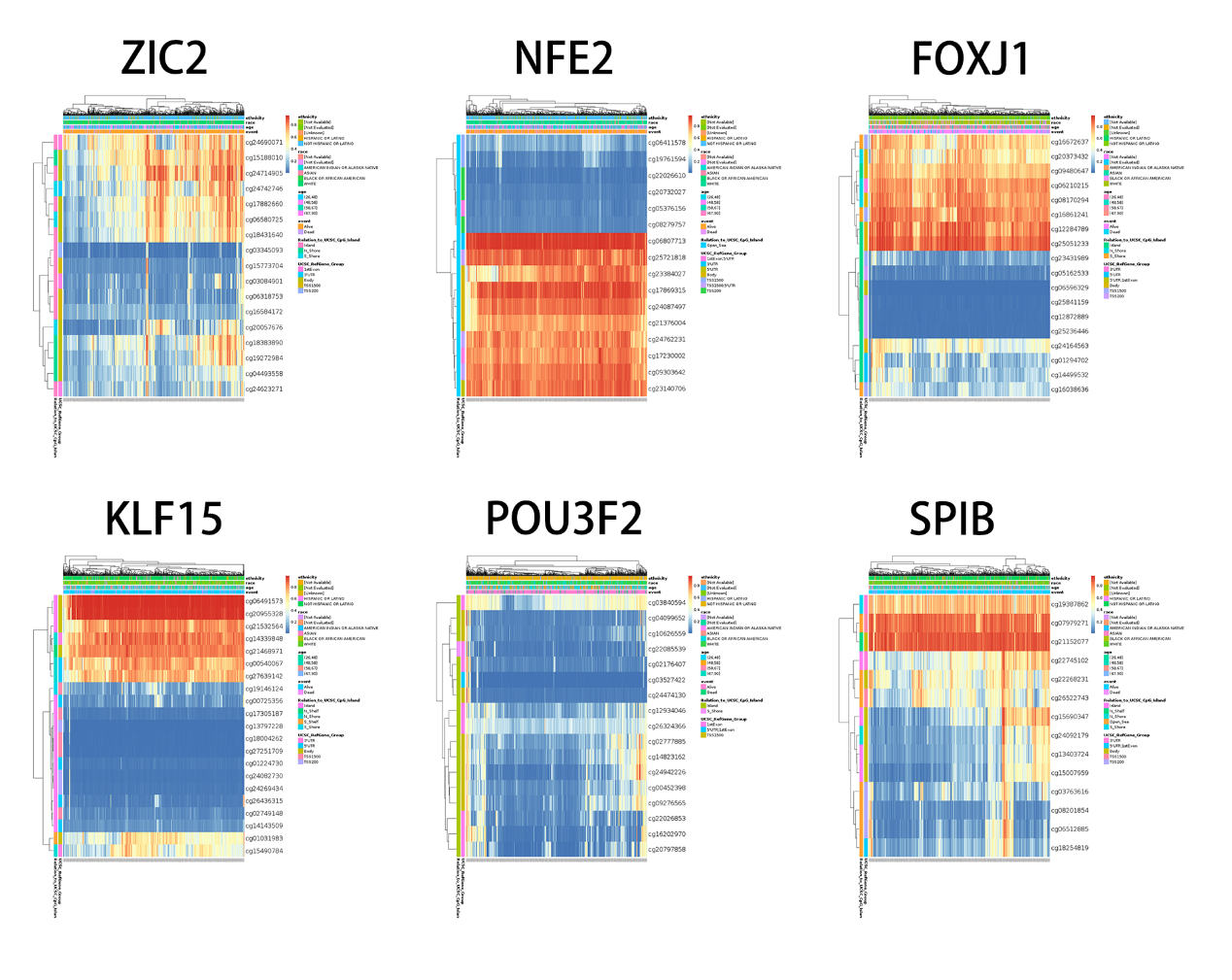
**

**Heatmap of DNA methylation clustering the expression levels of the 6 SERGs in BRCA**

**Prognostic Value of Single CpG of the SERGs in BRCA by MethSurv platform.**

| **Gene-CpG** | **HR** | **LR Test p-value** |
| --- | --- | --- |
| **NFE2-3’UTR-Open_Sea-cg06807713** | **0.049*** | **0.634 (0.41-0.981)** |
| **NFE2-TSS1500;5’UTR-Open_Sea-cg25721818** | **0.043*** | **1.521(1.005-2.303)** |
| **NFE2-Body-Open_Sea-cg23384027** | **0.047*** | **0.67 (0.452-0.995)** |
| **NFE2-Body-Open_Sea-cg17869315** | **0.0029*** | **0.485 (0.29-0.81)** |
| **NFE2-Body-Open_Sea-cg24087497** | **0.00048*** | **0.439 (0.295-0.653)** |
| **FOXJ1-TSS1500-S_Shore-cg16861241** | **0.023*** | **0.636 (0.431-0.938)** |
| **FOXJ1-Body-Island-cg12284789** | **0.017*** | **0.59 (0.388-0.897))** |
| **FOXJ1-Body-Island-cg25051233** | **0.0091*** | **0.569 (0.377-0.857)** |
| KLF15-Body-Island-cg06491573 | 0.05 | 1.525 (0.987-2.357) |
| KLF15-Body-Island-cg20955328 | 0.21 | 1.336 (0.84-2.126) |
| SPIB-TSS1500-Open_Sea-cg07979271 | 0.35 | 0.831 (0.563-1.226) |
| SPIB- Body-N_Shelf-cg21152077 | 0.16 | 0.73 (0.475-1.124) |

The threshold of significance was LR Test p-value<0.05.
