# Supplementary material for: A novel super-enhancer-related gene signature predicts prognosis and immune microenvironment for breast cancer
Source: BMC Cancer. 2023 Aug 18;23:776. doi: 10.1186/s12885-023-11241-2 (PMC10439574; doi:10.1186/s12885-023-11241-2)
Supplement: Supplementary file 7 — Supplementary Material 7 [file 12885_2023_11241_MOESM7_ESM.docx]

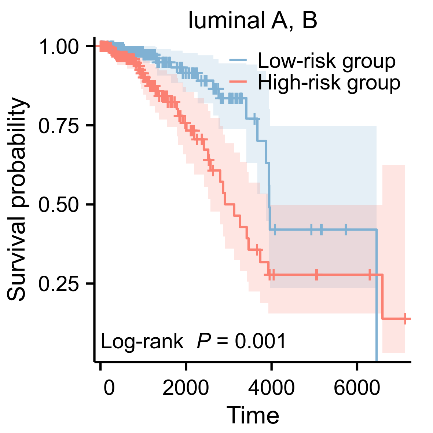

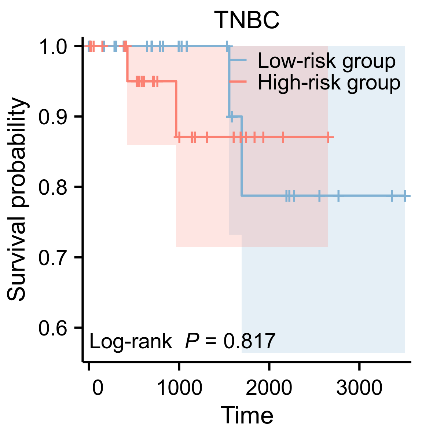


**KM curves for OS prediction in subgroups of luminal A, B breast cancer and triple negative breast cancer (TNBC).**
